# RBM3 promotes neurogenesis in a niche-dependent manner via IMP2-IGF2 signaling pathway after hypoxic-ischemic brain injury

**DOI:** 10.1038/s41467-019-11870-x

**Published:** 2019-09-04

**Authors:** Xinzhou Zhu, Jingyi Yan, Catherine Bregere, Andrea Zelmer, Tessa Goerne, Josef P. Kapfhammer, Raphael Guzman, Sven Wellmann

**Affiliations:** 1 University Children’s Hospital Basel (UKBB), University of Basel, Basel, 4056 Switzerland; 2 Department of Biomedicine, University of Basel, Basel, 4056 Switzerland; 3 Department of Neurosurgery, University Hospital Basel, Basel, 4031 Switzerland; 4 University Children’s Hospital Regensburg (KUNO), University of Regensburg, Regensburg, 93053 Germany

**Keywords:** Adult neurogenesis, Molecular medicine

## Abstract

Hypoxic ischemia (HI) is an acute brain threat across all age groups. Therapeutic hypothermia ameliorates resulting injury in neonates but its side effects prevent routine use in adults. Hypothermia up-regulates a small protein subset that includes RNA-binding motif protein 3 (RBM3), which is neuroprotective under stressful conditions. Here we show how RBM3 stimulates neuronal differentiation and inhibits HI-induced apoptosis in the two areas of persistent adult neurogenesis, the subventricular zone (SVZ) and the subgranular zone (SGZ), while promoting neural stem/progenitor cell (NSPC) proliferation after HI injury only in the SGZ. RBM3 interacts with *IGF2* mRNA binding protein 2 (IMP2), elevates its expression and thereby stimulates IGF2 release in SGZ but not SVZ-NSPCs. In summary, we describe niche-dependent regulation of neurogenesis after adult HI injury via the novel RBM3-IMP2-IGF2 signaling pathway.

## Abstract (web summary)

Therapeutic hypothermia is a potent tool in the treatment of neonatal hypoxic-ischemic (HI) injury, yet the underlying mechanisms remain unclear. Here, authors demonstrate how the RNA-binding motif protein RBM3, which is induced by mild cooling while global translation rate is slowed down, contributes substantially to neuroregeneration after adult HI injury, specifically in the subventricular zone and subgranular zone.

## Introduction

The brain is the organ most vulnerable to oxygen and energy deprivation. Hypoxic ischemia (HI) inflicts devastating brain injury across all age groups. Birth asphyxia is the leading cause of neonatal encephalopathy and lifelong neurologic sequelae^1^. In older children and adults, out-of-hospital cardiac arrest is the main cause of global HI brain injury and subsequent morbidity and mortality^2^.

Mild hypothermia (32–34 °C) significantly reduces the risk of death and disability from birth asphyxia^1,3^, but has side effects that complicate its use in adults after brain injury^4,5^. Unraveling the cellular and molecular mechanisms of the neuroprotection conferred by hypothermia may therefore help the development of targeted treatment.

As well as slowing the cellular metabolic rate, mild hypothermia induces a subset of stress-response proteins in mammals, including RNA-binding motif protein 3 (RBM3)^6^. Previous reports from our group and others showed RBM3 to be neuroprotective under various stressful conditions through diverse cellular and molecular mechanisms, such as maintaining synapse plasticity via reticulon-3 (RTN3), suppressing poly (ADP-ribose) polymerase (PARP) cleavage, and inhibiting endoplasmic reticular (ER) stress-induced apoptosis^7–10^.

Rodent studies have shown that RBM3 is highly expressed in proliferating and differentiating brain regions such as the subventricular zone (SVZ), the rostral migratory stream (RMS), and the subgranular zone (SGZ) of the dentate gyrus (DG) in both neonates and adults^9,11^. Notably, RBM3 colocalizes with neural stem cell marker nestin^11^ and neuroblast marker doublecortin (Dcx)^9^, indicating its role in maintaining neural stem/progenitor cell (NSPC) self-renewal and neurogenesis.

In adults, the SVZ and SGZ are the only two well-characterized neurogenic niches not only in rodents but also in humans^12–14^. NSPC proliferation and neurogenesis are stimulated in both niches after ischemic injury in order to aid post-ischemic recovery^15^. However, it remains uncertain whether hypothermia promotes neuroregeneration after HI injury, as different cooling settings and injury models produce conflicting conclusions^16^. We focused exclusively on the effects of RBM3 on NSPC proliferation and neurogenesis in the SVZ and SGZ niches after HI injury in vitro and in vivo.

## Results

### RBM3 stimulates NSPC proliferation in SGZ but not in SVZ

As observed in other studies, RBM3 knockout (KO) mice do not exhibit an obvious phenotype under physiological conditions^17^. However, under pathological conditions such as neurodegenerative disease, RBM3 acts as a stress-response protein, significantly influencing neural survival and function^7^. In studying its role in adult neurogenesis we found no difference in brain weight between adult RBM3 KO and wild-type (WT) mice raised under normal conditions (Supplementary Fig. 1a). Nor did we detect structural abnormalities in the brains or any difference in the SVZ and DG volumes of KO compared to WT mice (Supplementary Fig. 1b).

Previously we and others had identified RBM3 in adult NSPCs and neuroblasts^9,11^. The present study confirmed its expression in sex determining region Y-box 2 (Sox2)+ NSPCs and Dcx + neuroblasts in both the SVZ and SGZ in vivo (Supplementary Fig. 1c, d). We monitored basal NSPC proliferation by injecting adult mice intraperitoneally with bromodeoxyuridine (BrdU) every other day for 7 days before sacrifice. The total number and density of proliferating (BrdU+ Sox2+) NSPCs in KO mice was similar to that in WT mice in both the SVZ and SGZ niches (Supplementary Fig. 1e, f). Further analysis by neurosphere assay revealed that the number and size of primary and secondary neurospheres isolated from KO mice resembled those in WT mice, regardless of origin (Supplementary Fig. 1g, h). Thus our data indicate that neurogenic potential appears unimpaired in adult RBM3-deficient mice under physiological growing conditions.

**Fig. 1:**
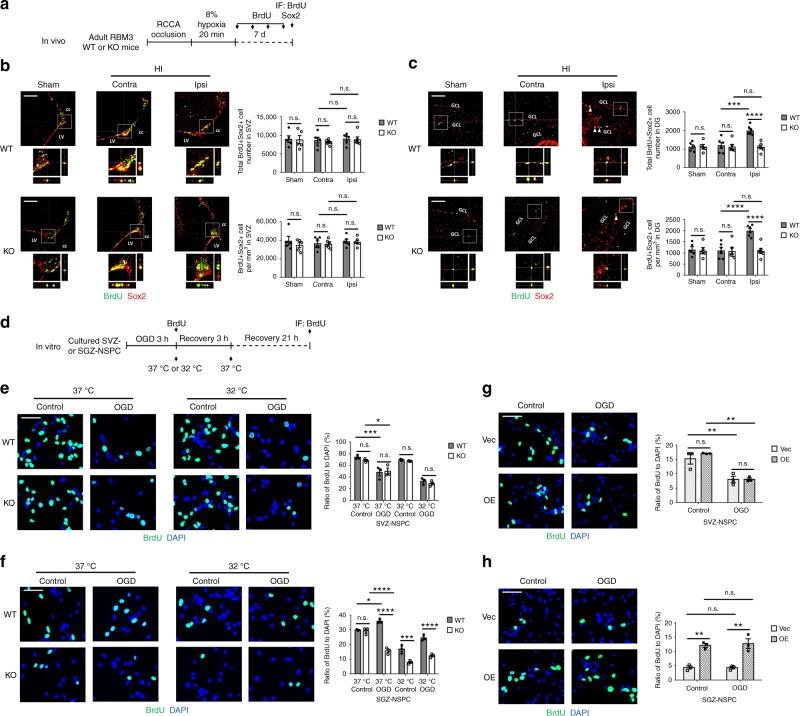
RBM3 is required for HI-induced proliferation of SGZ- but not SVZ-NSPC in vivo and in vitro. **a** Illustration of in vivo HI model and analysis of NSPC proliferation. RCCA right common carotid artery, IF immunofluorescence. **b**, **c** Representative immunofluorescent staining of BrdU and Sox2 in SVZ (**b**) and DG (**c**) of RBM3 WT and KO animals treated with HI and recovered for 7 days with BrdU injection every other day. Animals in control group received sham surgery. Orthogonal view confirmed the colocalization of BrdU and Sox2. BrdU+ Sox2+ cell number and density in the SVZ or DG were estimated. Five sham animals and six HI animals were counted per group (Sham: *n* = 5, HI: *n* = 6). Sham, sham group; contra, contralateral (uninjured side) in HI group; ipsi, ipsilateral (injured side) in HI group. Scale bar: 100 µm. LV lateral ventricle, cc corpus callosum, GCL granular cell layer. Repeated measures two-way ANOVA was used for statistical analysis; n.s. not significant; ***p < 0.001; ****p < 0.0001. **d** Illustration of in vitro OGD model and analysis of NSPC proliferation. OGD oxygen-glucose deprivation, IF immunofluorescence. Only WT or KO NSPCs underwent hypothermic treatment (32 °C). Plasmid-transfected NSPCs were always cultured at 37 °C. **e**–**h** Representative immunofluorescent staining of BrdU and DAPI in NSPCs after OGD stress. SVZ-NSPCs (**e**) and SGZ-NSPCs (**f**) from RBM3 WT or KO mice were treated with OGD and reoxygenated in BrdU-containing medium at 37 or 32 °C for 3 h, followed by 37 °C for an additional 21 h. The ratio of BrdU+/DAPI+ cells were quantified (three independent experiments, *n* = 3). Three-way ANOVA was used for statistical analysis; n.s. not significant; *p < 0.05; ***p < 0.001; ****p < 0.0001. SVZ-NSPCs (**g**) and SGZ-NSPCs (**h**) transfected with empty vector (Vec) or RBM3 overexpression (OE) plasmid were treated with OGD and reoxygenated in BrdU-containing medium at 37 °C for 24 h. The ratio of BrdU+/DAPI+ cells was quantified (three independent experiments, *n* = 3). Scale bar: 50 µm. Two-way ANOVA was used for statistical analysis; n.s. not significant; **p < 0.01. All data are presented as mean ± SEM

Next, we applied an acute brain HI model to adult RBM3 WT and KO mice to determine whether RBM3 depletion affects NSPC proliferation and neurogenesis under pathological conditions. After permanent ligation of the right common carotid artery, the animals were subjected to 8% hypoxia for 20 min, followed by recovery for 7 days with repeated BrdU pulsing (Fig. 1a). We used equation (1) in Methods to estimate infarction volume. Infarction volume was significantly greater in KO than in WT brains (Supplementary Fig. 2a). Regardless of more neuronal loss in KO mice, the volumes of both SVZ and DG did not differ between ipsilateral and contralateral sides in WT and KO mice (Supplementary Fig. 2b). Equation (2) in Methods was used for stereological cell quantification. After 7 days recovery from HI, total BrdU+ cell density in the SVZ and DG were both increased in the ipsilateral side of WT and KO mice compared to contralateral side, but no difference was observed between WT and KO mice (Supplementary Fig. 2c, d). Simultaneously, large quantities of reactive (glial fibrillary acidic protein [GFAP]+) astrocytes and (ionized calcium-binding adapter molecule 1 [Iba1]+) microglia were induced in the SVZ and surrounding areas such as striatum and corpus callosum, as well as in the ipsilateral hippocampus adjacent to the ischemic core, in both WT and KO brains after HI injury (Supplementary Fig. 3a, b). We saw evidence of oligodendrocyte transcription factor 2 (Olig2)+ oligodendrocyte precursor cell stimulation in the ipsilateral corpus callosum and DG of WT mice but not KO mice (Supplementary Fig. 3c). We observed more terminal deoxynucleotidyl transferase dUTP nick end labeling (TUNEL)+ apoptotic cells in the ischemic cores in ipsilateral cortex and striatum in KO brains than in WT brains (Supplementary Fig. 3d). The total number and density of newborn (BrdU+ Sox2+) NSPCs were barely affected in the SVZ of WT and KO mice (Fig. 1b). Strikingly, whereas there was a significant increase of BrdU+ Sox2+ NSPCs in the HI ipsilateral SGZ of WT mice compared to HI contralateral and sham animals, in KO littermates, BrdU+ Sox2+ NSPCs were not induced in the SGZ (Fig. 1c). Albeit we found some BrdU+ cells in the SGZ of KO mice, many of them were Sox2-negative cells, indicating discrepancies between neurogenic niches in RBM3 effect on NSPC proliferation.

To characterize the role of RBM3 in NSPC proliferation in vitro, we isolated and cultured nestin+ Sox2+ NSPCs from the SVZ and SGZ of RBM3 WT and KO mice (Supplementary Fig. 4a). Cultured NSPCs were challenged with oxygen-glucose deprivation (OGD), an in vitro model of HI, before being reoxygenated for 24 h in BrdU-containing complete medium (Fig. 1d). NSPC proliferation from the SVZ was virtually similar after OGD between WT and KO cultures. In contrast, NSPC proliferation from the SGZ was dramatically elevated in WT cultures but reduced in KO cultures (Fig. 1e, f), consistent with the in vivo findings (Fig. 1b, c). In addition, we treated NSPCs after OGD with mock stress or hypothermia for 3 h (Fig. 1d), thereby enhancing RBM3 expression (Supplementary Fig. 4b). Overall, hypothermia inhibited cell proliferation, but more so in KO than in WT SGZ-NSPCs (Fig. 1f). We detected no difference in RBM3-depleted SVZ-NSPCs (Fig. 1e). Furthermore, when we overexpressed recombinant RBM3 in SVZ- and SGZ-NSPCs after OGD (Supplementary Fig. 4c), only the SGZ-NSPCs proliferated at a higher rate than in a mock vector group (Fig. 1g, h).

Taken together, our data suggest that while RBM3 does not affect basal NSPC proliferation under normal growing conditions, after HI injury it stimulates the proliferation of SGZ-NSPCs but not SVZ-NSPCs in vivo and in vitro.

### RBM3 promotes NSPC neuronal differentiation in SVZ and SGZ

We next examined whether RBM3 regulates NSPC differentiation and contributes to neurogenesis.

**Fig. 2:**
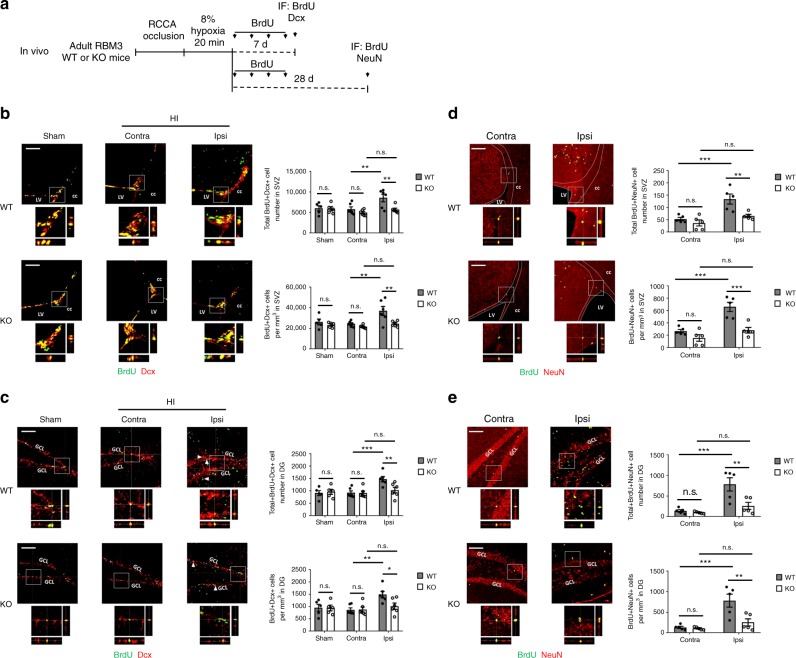
RBM3 promotes neurogenesis in both SVZ and SGZ after HI injury in vivo. **a** Illustration of in vivo HI model and analysis of neurogenesis. RCCA right common carotid artery, IF immunofluorescence. **b**, **c** Representative immunofluorescent staining of BrdU and Dcx in SVZ (**b**) or DG (**c**) of RBM3 WT and KO animals treated with HI and recovered for 7 days with BrdU injection every other day. Animals in control group received sham surgery. Orthogonal view confirmed the co-localization of BrdU and Dcx. BrdU+ Dcx+ cell number and density in the SVZ or DG were estimated. Five sham animals and six HI animals were counted per group (Sham: *n* = 5, HI: *n* = 6). Sham, sham group; contra, contralateral (uninjured side) in HI group; ipsi, ipsilateral (injured side) in HI group. Scale bar: 100 µm. LV lateral ventricle, cc corpus callosum, GCL granular cell layer. Repeated measures two-way ANOVA was used for statistical analysis; n.s. not significant; *p < 0.05; **p < 0.01; ***p < 0.001. **d**, **e** Representative immunofluorescent staining of BrdU and NeuN in SVZ (**d**) or DG (**e**) of RBM3 WT and KO animals treated with HI and recovered for 28 days with BrdU injection every 2 days in the first week of recovery. Orthogonal view confirmed the colocalization of BrdU and NeuN. BrdU+ NeuN+ cell number and density in the SVZ or DG were estimated. Five animals were counted per group (*n* = 5). Contra, contralateral (uninjured side); ipsi, ipsilateral (injured side). Scale bar: 100 µm. LV lateral ventricle, cc corpus callosum, GCL granular cell layer. Repeated measures two-way ANOVA was used for statistical analysis; n.s. not significant; **p < 0.01; ***p < 0.001. All data are presented as mean ± SEM

In vivo, after 7 days recovery from HI treatment (Fig. 2a), newborn (BrdU+ Dcx+) neuroblasts showed significant stimulation in the HI ipsilateral SVZ and SGZ of WT mice compared to the HI contralateral and sham group (Fig. 2b, c). In RBM3-depleted mice, on the other hand, newborn neuroblast stimulation was absent in the ipsilateral SVZ and SGZ (Fig. 2b, c). In long-term studies, researchers have found that most newborn NSPCs die before maturation, with the remaining cells differentiating into mature neurons as an inadequate repair of the damaged brain tissue^18,19^. After a longer recovery period (28 days) after HI injury (Fig. 2a), only a few BrdU+ cells survived in SVZ but more BrdU+ cells survived in DG (Supplementary Fig. 2e, f). A significantly higher number and density of total BrdU+ cells in the SVZ and DG was found in WT mice compared to KO mice (Supplementary Fig. 2e, f). Furthermore, we found increased numbers of mature BrdU+ NeuN+ neurons in both the ipsilateral SVZ and DG in WT mice (Fig. 2d, e) but reduced numbers in KO mice (Fig. 2d, e).

**Fig. 3:**
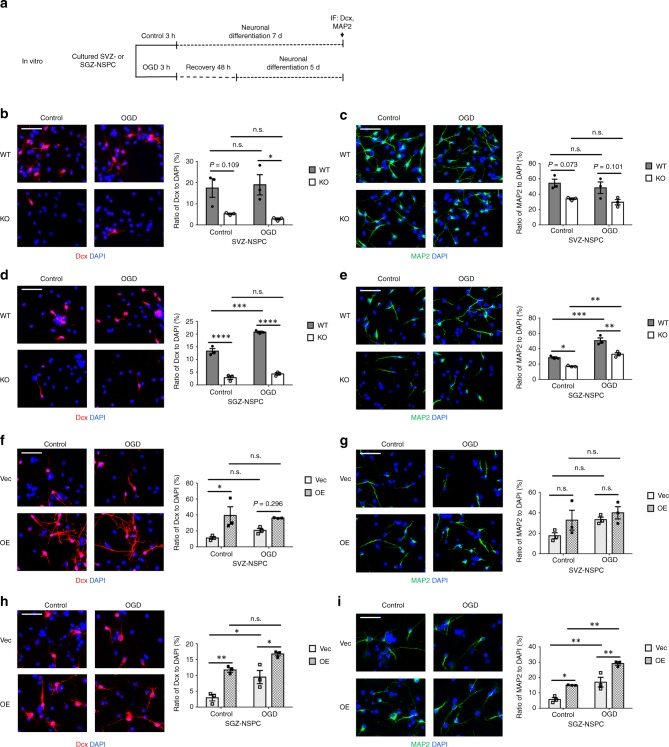
RBM3 promotes neuronal differentiation of both SVZ-NSPC and SGZ-NSPC after OGD in vitro. **a** Illustration of in vitro neuronal differentiation assay. NSPCs were cultured in neuronal differentiation medium for 7 days (control), or first challenged with OGD and then reoxygenated in NSPC complete culture medium for the first 2 days, followed by switching to neuronal differentiation medium for 5 days (OGD). OGD oxygen-glucose deprivation, IF immunofluorescence. **b**–**i** RBM3 WT and KO NSPCs from SVZ (**b**, **c**) or SGZ (**d**, **e**), and WT NSPCs transfected with empty vector (Vec) or RBM3 overexpressing vector (OE) with SVZ (**f**, **g**) or SGZ (**h**, **i**) origins were used in neuronal differentiation assay. Neuroblast marker Dcx (**b**, **d**, **f**, **h**) and neuronal marker MAP2 (**c**, **e**, **g**, **i**) were stained and the ratio of immunoreactive cells to DAPI-positive cells was calculated (three independent experiments, *n* = 3). Representative images were presented and statistical data were acquired. Scale bar: 50 µm. Two-way ANOVA was used for statistical analysis; n.s. not significant; *p < 0.05; **p < 0.01; ***p < 0.001; ****p < 0.0001. All data are presented as mean ± SEM

In vitro, we differentiated NSPCs into neurons by culture in growth factor-free medium for 7 days. At the same time, in order to assess the impact of OGD on differentiation, we treated NSPCs with OGD followed by reoxygenation for 7 days: in normal NSPC culture medium for the first 2 days, to allow enough time for activating downstream signaling in stem cell status, and in growth factor-free neuronal differentiation medium the remaining 5 days (Fig. 3a).

In this neuronal differentiation assay, OGD treatment induced a remarkable increase in the ratio of Dcx+ neuroblasts and microtubule-associated protein 2 (MAP2)+ neurons to all 4’, 6-diamidino-2-phenylindole (DAPI)+ cells in SGZ-NSPCs (Fig. 3d, e) but not in SVZ-NSPCs (Fig. 3b, c). In the absence of RBM3, the percentages of both differentiated neuroblasts and neurons remained nearly unchanged in SVZ-NSPCs (Fig. 3b, c) while decreasing significantly in SGZ-NSPCs (Fig. 3d, e). When RBM3 was overexpressed, on the other hand, neuroblast and neuron percentages remained almost unchanged in SVZ-NSPCs (Fig. 3f, g) while increasing significantly in SGZ-NSPCs (Fig. 3h, i).

Similarly, we differentiated NSPCs into astrocytes and oligodendrocytes with or without OGD treatment (Supplementary Fig. 5a). We used S100 to label glial precursors. The commonly used astrocyte marker GFAP was expressed in all the differentiated cells, and was also present in undifferentiated NSPCs (Supplementary Fig. 5b) as reported in other publications^20,21^, thus not used. We found no difference when RBM3 expression was altered in an astrocyte differentiation assay (Supplementary Fig. 5c-f).

In an oligodendrocyte differentiation assay we used Olig2 to label oligodendrocyte precursor cells (OPCs), but not oligodendrocyte marker maltose binding protein (MBP) as there were only a few MBP+ cells (<1%) after 7 days of differentiation (Supplementary Fig. 5g). In contrast to astrocyte differentiation, OGD treatment induced OPC differentiation in both SVZ- and SGZ-NSPCs (Supplementary Fig. 5h-k). However, although RBM3 appeared to promote OPC differentiation in the absence of OGD, we found little difference in Olig2+ cell percentages after OGD when changing the level of RBM3 expression (Supplementary Fig. 5h-k).

In summary, we observed induction of neurogenesis after HI injury. Our results support the notion that RBM3 enhances neuronal differentiation potential in both neurogenic niches. However, as RBM3 has less influence on the neuronal differentiation of SVZ-NSPCs than of SGZ-NSPCs, we can anticipate a smaller impact on SVZ neurogenesis. RBM3 expression has no notable effect on glial cell differentiation.

### RBM3 limits HI-induced apoptosis in both SVZ- and SGZ-NSPCs

**Fig. 4:**
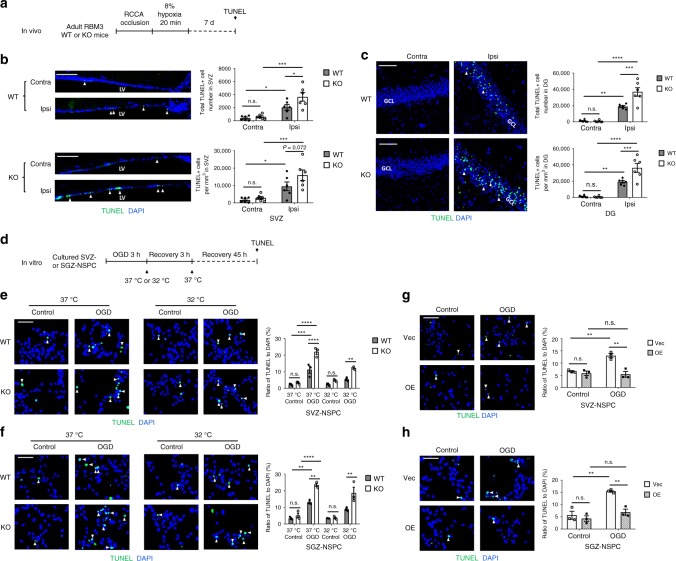
RBM3 prevents HI-induced apoptosis in vivo and in vitro. **a** Illustration of in vivo HI model and analysis of apoptosis. RCCA right common carotid artery, TUNEL terminal deoxynucleotidyl transferase dUTP nick end labeling. **b**, **c** Representative immunofluorescent TUNEL staining in SVZ (**b**) or DG (**c**) of RBM3 WT and KO animals treated with HI and recovered for 7 days. Total TUNEL+ cell number and density in the SVZ or DG were estimated. Six animals were counted per group (*n* = 6). Scale bar: 50 µm. Contra contralateral (uninjured side), Ipsi ipsilateral (injured side), LV lateral ventricle, GCL granular cell layer. Repeated measures two-way ANOVA was used for statistical analysis; n.s. not significant; *p < 0.05; **p < 0.01; ***p < 0.001; ****p < 0.0001. **d** Illustration of in vitro OGD model and analysis of apoptosis. OGD oxygen-glucose deprivation. Only WT or KO NSPCs underwent hypothermic treatment (32 °C). Plasmid-transfected NSPCs were always cultured at 37 °C. **e**–**h** Representative immunofluorescent TUNEL in NSPCs after OGD stress. SVZ-NSPCs (**e**) and SGZ-NSPCs (**f**) from RBM3 WT or KO mice were treated with OGD and hypothermia as stated in Fig.1 legend, except for 48 h reoxygenation period instead of 24 h in total. The ratio of TUNEL+/DAPI+ cells was quantified (three independent experiments, *n* = 3). Three-way ANOVA was used for statistical analysis; n.s. not significant; **p < 0.01; ***p < 0.001; ****p < 0.0001. SVZ-NSPCs (**g**) and SGZ-NSPCs (**h**) transfected with empty vector (Vec) or RBM3 overexpression (OE) plasmid were treated with OGD and reoxygenated at 37 °C for 48 h. The ratio of TUNEL+/DAPI+ cells was quantified (three independent experiments, *n* = 3). Scale bar: 50 µm. Two-way ANOVA was used for statistical analysis; n.s. not significant; **p < 0.01. All data are presented as mean ± SEM

To exclude the possibility that the discrepancy in proliferation rates between SVZ- and SGZ-derived NSPCs resulted from different severities of HI-induced apoptosis, we performed TUNEL staining (Fig. 4a) and found significantly more apoptotic cells at the ipsilateral side in RBM3 KO mice than in their WT littermates in both stem cell niches, in the lateral wall (Fig. 4b) and entire DG (Fig. 4c). Only few apoptotic cells were observed in the lateral tail of the SVZ (Supplementary Fig. 3d). In contrast, very few TUNEL+ cells were present at the contralateral sides in either RBM3 KO or WT HI animals (Fig. 4b, c).

To assess the anti-apoptotic effect of RBM3 in vitro, we quantified TUNEL+ cells 48 h after OGD (Fig. 4d). OGD challenge induced significant apoptosis in both SVZ- and SGZ-derived NSPCs (Fig. 4e, f). Absence of RBM3 clearly exacerbated post-OGD apoptosis (Fig. 4e, f). Hypothermia attenuated apoptosis in WT NSPCs, but only marginally in KO NSPCs, suggesting that RBM3 partially mediates hypothermic cytoprotection in NSPCs (Fig. 4e, f). On the other hand, forced RBM3 expression blocked OGD-induced apoptosis (Fig. 4g, h).

Our results suggest that RBM3 limits HI-induced apoptosis overall in vivo and in vitro thus at least partially mediating the protective effects of cooling. However, the protective effect applies to both SVZ- and SGZ-NSPCs, indicating that the discrepancy in post-OGD proliferation is not due to apoptosis.

### RBM3-IMP2-IGF2 axis mediates niche-dependent proliferation

To unravel the molecular mechanism of reduced proliferation in RBM3-depleted hippocampal NSPC, we performed RNA sequencing (RNA-seq) to identify transcriptome changes in RBM3 KO mice. We used postnatal day 3 (P3) and 2–3 month adult hippocampi from RBM3 WT and KO mice without HI injury. The quality of extracted total RNA fulfilled the requirements for RNA-seq (Supplementary Data 1). As expected, rare global differences of gene expression were seen when comparing RBM3 WT and KO even when we applied a less stringent cutoff condition (Supplementary Fig. 6a-c, Supplementary Data 2 and 3), consistent with the unaltered phenotype in physiological condition (Supplementary Fig. 1a, b, e-h). Due to the limited number of differentially expressed genes (DEGs), Gene Set Enrichment Analysis (GSEA) provided limited information (Supplementary Fig. 6d). Among the few common DEGs between P3 and adult lists, we identified the transcript of *insulin-like growth factor 2* (*IGF2)* as one of the candidates, which was downregulated when RBM3 was absent (Supplementary Fig. S6a-S6c, Supplementary Data 2 and 3). At the same time, we identified candidate *IGF2* mRNA binding proteins (IMPs) from our previously published screening list of RBM3 interactors^10^, known to regulate *IGF2* mRNA stability and promote its expression^22^. Based on these two independent screening approaches we focused on this IGF as in addition it had been reported to induce niche-dependent proliferation of adult NSPCs^23,24^. Consistent with previous publications^25,26^, we found all three IMPs to be expressed at much lower levels in adult NSPCs than in NSPCs from postnatal day 0 (P0) mice (Supplementary Fig. 7a). *IMP1* expression was almost undetectable, while *IMP3* expression was much lower than that of *IMP2* in WT adult NSPCs (Supplementary Fig. 7a). Given additional evidence that IMP2 promotes neuronal differentiation in embryonic neocortical NSPCs^27^, we tested the hypothesis that RBM3 regulates NSPC proliferation and may involve IMP2-IGF2 signaling in adult NSPCs.

**Fig. 5:**
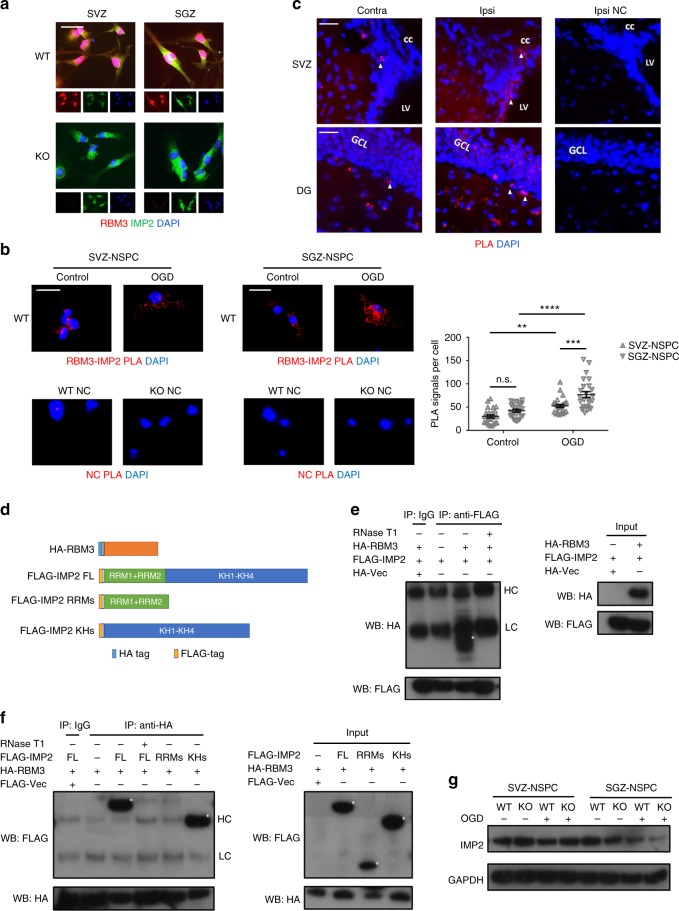
RBM3 interacts with IMP2. **a** Representative immunofluorescent staining of RBM3 and IMP2 in SVZ-NSPCs and SGZ-NSPCs from adult WT mouse brain. RBM3 (red), IMP2 (green) and DAPI (blue) were merged. Scale bar: 25 µm. **b** Representative immunofluorescent images from proximity ligation assay. SVZ-NSPCs and SGZ-NSPCs were challenged with OGD and reoxygenated for 3 h. WT NSPCs omitting primary antibodies (WT NC) or KO NSPCs served as negative controls (KO NC). Fluorescent dots indicating single RBM3-IMP2 interactions were counted in each cell, and 25 cells per group were used for quantification (*n* = 25). RBM3-IMP2 PLA signals (red) and DAPI (blue) were merged. Scale bar: 25 µm. Two-way ANOVA was used for statistical analysis; n.s. not significant; **p < 0.01; ***p < 0.001; ****p < 0.0001. All data are presented as mean ± SEM. **c** Representative image of proximity ligation assay of RBM3 and IMP2 in frozen brain sections from adult mice treated with HI and 7 days recovery. Scale bar: 50 µm. Contra contralateral (uninjured side), ipsi ipsilateral (injured side), LV lateral ventricle, cc corpus callosum, GCL granular cell layer, NC negative control without primary antibodies. **d** Schematic illustration of RBM3 and IMP2 constructs. Full-length (FL) RBM3 was fused with an N-terminal HA tag. Full-length (FL) IMP2 was truncated to N-terminal domain with two RNA-recognition motifs (RRMs) or C-terminal domain with four K Homology motifs (KHs); all of the constructs included an N-terminal FLAG tag. **e**, **f** Representative co-immunoprecipitation graphs of full-length or truncated RBM3 and IMP2 in HEK293 cells. Full-length FLAG-IMP2 was co-overexpressed with vector (Vec, negative control), or HA-RBM3 with or without RNase T1 pre-treatment. FLAG antibody was used to precipitate FLAG-IMP2. FLAG-IMP2 and HA-RBM3 in input or IP samples were detected by anti-FLAG or anti-HA antibodies using Western blot (**e**). A reciprocal CoIP was performed using full-length HA-RBM3 to precipitate full-length FLAG-IMP2, FLAG-RRMs or FLAG-KHs (**f**). Asterisks indicate target bands. HC heavy chain of IgG used for immunoprecipitation, LC light chain of IgG used for immunoprecipitation. **g** Representative Western blot of IMP2 expression in SVZ-NSPC and SGZ-NSPC after mock or OGD treatment in the presence or absence of RBM3

First we examined RBM3-IMP2 interaction in NSPCs. In cultured NSPCs, RBM3 was expressed predominantly in nuclei but also in cytoplasm, while IMP2 expression was confined to cytoplasm (Fig. 5a). Proximity ligation assay showed that RBM3 and IMP2 were adjacent in both SVZ and SGZ-NSPCs, while OGD treatment significantly increased the number of positive signals per cell, indicating more RBM3-IMP2 interactions responding to OGD (Fig. 5b). Additionally, RBM3-IMP2 interactions were more abundant in SGZ-NSPCs than those in SVZ-NSPCs after OGD (Fig. 5b). In the SVZ and SGZ regions in vivo, RBM3 and IMP2 were co-expressed (Supplementary Fig. 7b) and showed adjacent localization in situ (Fig. 5c).

In HEK293 cells where we first identified RBM3-IMP2 interaction^10^, and in NSPCs from P0 mouse brain, RBM3 and IMP2 expressions were both at high level (Supplementary Fig. 7c) and their interactions were much more abundant (Supplementary Fig. 7d), compared to adult NSPCs (Fig. 5b). Endogenous RBM3-IMP2 interactions were confirmed by co-immunoprecipitation (CoIP) directly in HEK293 cells and P0 NSPCs (Supplementary Fig. 7e). We further expressed recombinant RBM3 and IMP2 in HEK293 cells and examined, which domains were required for their interaction by using CoIP. IMP2 is known to contain two RNA-binding motifs (RRMs) and four K Homology (KH) domains, which can all bind RNA, but previous reports identified only the KH domains, and not the RRM domains as directly mediating *IGF2* mRNA binding^28^. To check which domains of IMP2 were required for RBM3-IMP2 interaction, we co-overexpressed full-length IMP2, truncated IMP2 RRMs (two RRM domains), and truncated IMP2 KHs (four K-homology domains) together with full-length RBM3 (Fig. 5d). The CoIP results indicated that the RBM3-IMP2 interaction was RNA-dependent because it was abolished by RNase treatment (Fig. 5e, f). As expected, only the KH domains and not the RRM domains, were essential for interactions with RBM3 (Fig. 5f), consistent with the finding that interaction is mediated by RNA.

**Fig. 6:**
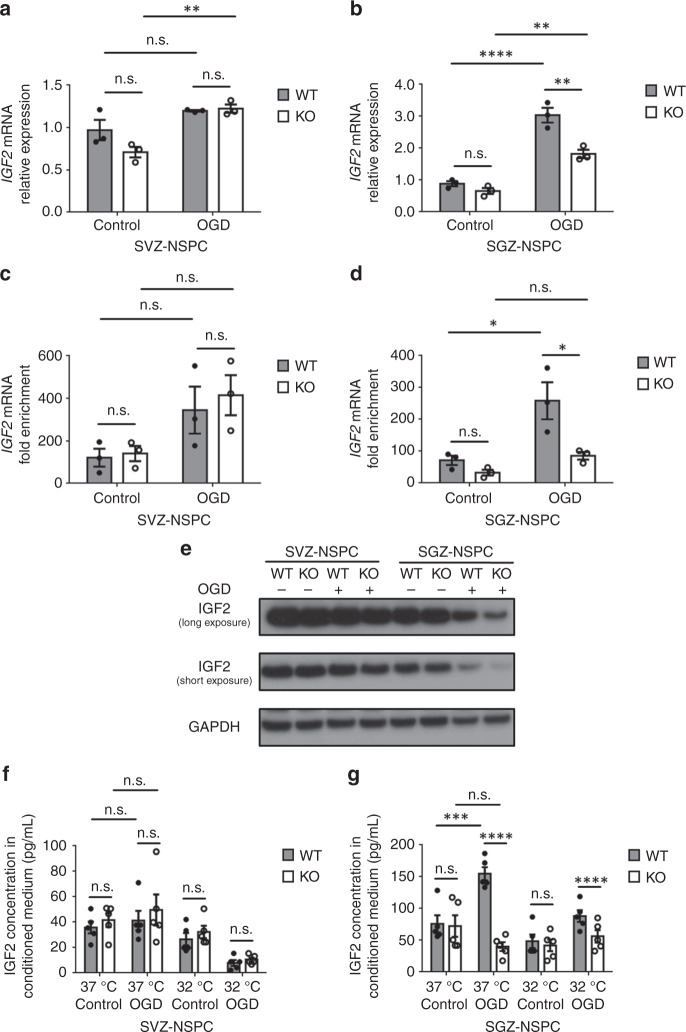
RBM3 regulates IGF2 expression in SGZ- but not SVZ-NSPCs in vitro. **a**, **b** Quantitative RT-PCR of *IGF2* mRNA expression in SVZ-NSPC (**a**) and SGZ-NSPC (**b**) after 3 h OGD and 3 h reoxygenation in the presence or absence of RBM3 (three independent experiments, *n* = 3). *IGF2* mRNA level was normalized to *GAPDH* by 2^−ΔΔCT^ method. Two-way ANOVA was used for statistical analysis; n.s. not significant; **p < 0.01; ****p < 0.0001. **c**, **d** The abundance of bound *IGF2* mRNA to IMP2 protein in RBM3 WT or KO NSPCs after 3 h OGD and 3 h reoxygenation. Lysates from SVZ-NSPC (**c**) or SGZ-NSPC (**d**) were subjected to RNA-immunoprecipitation. Anti-IMP2 antibody was used to precipitate IMP2 protein, and the amount of bound *IGF2* mRNA was detected by quantitative RT-PCR (three independent experiments, *n* = 3). Normal mouse immunoglobulin (msIgG) was served as negative control in immunoprecipitation. *IGF2* mRNA level was normalized to input by 2^−ΔΔCT^ method, and fold enrichment was normalized to msIgG group which was set as 1. Two-way ANOVA was used for statistical analysis; n.s. not significant; *p < 0.05. **e** Representative Western blot of IGF2 expression in SVZ-NSPC and SGZ-NSPC after mock or OGD treatment in the presence or absence of RBM3. **f**, **g** IGF2 levels in culture medium by ELISA. SVZ-NSPCs (**f**) and SGZ-NSPCs (**g**) were stressed with OGD followed by reoxygenation at 37 or 32 °C for 3 h, then incubated at 37 °C for an additional 21 h. Culture medium was immediately collected for IGF2 measurement by ELISA (five independent experiments, *n* = 5). Three-way ANOVA was used for statistical analysis; n.s. not significant; ***p < 0.001; ****p < 0.0001. All data are presented as mean ± SEM

Having confirmed RBM3-IMP2 interaction, we wished to determine whether RBM3 regulates IMP2 and its downstream IGF2 expression. In whole brain, we detected slightly lower protein levels of IMP2 but not IGF2 in RBM3 KO mice (Supplementary 7f). In cultured NSPCs, we observed no difference in post-OGD IMP2 expression in SVZ-NSPCs, as opposed to a slight decrease in SGZ-NSPCs, and a further decrease when RBM3 was absent (Fig. 5g). In injured hemisphere, IMP2 was generally induced in GFAP+ astrocytes in both SVZ and adjacent striatum and in the entire DG (Supplementary Fig. 7g). Therefore we intended to figure out whether the downstream effector IGF2 changes in a niche-dependent manner. We detected increased *IGF2* mRNA expression in WT SGZ-NSPCs but not in SVZ-NSPCs after OGD in vitro, and less increase in KO SGZ-NSPCs (Fig. 6a, b). In addition, using RNA-immunoprecipitation, we identified more *IGF2* mRNA bound to IMP2 protein after OGD in WT SGZ-NSPCs and an upward trend in WT SVZ-NSPCs (Fig. 6c, d). In the absence of RBM3, enrichment of bound *IGF2* mRNA was clearly reduced in SGZ-NSPCs after OGD when comparing to WT, but unchanged in SVZ-NSPCs (Fig. 6c, d). Interestingly, endogenous IGF2 protein levels were reduced instead of enhanced after OGD in SGZ-NSPCs, probably due to increased secretion (Fig. 6e). When RBM3 was depleted, endogenous IGF2 protein decreased in SGZ-NSPCs after OGD (Fig. 6e). IGF2 protein levels in SVZ-NSPCs were not significantly affected by either OGD or RBM3 depletion, consistent with the unchanged *IGF2* mRNA data and the trend of IMP2 protein (Fig. 5g, 6c, e). On measuring the levels of IGF2 released into culture medium after OGD stress, we found those in SVZ-NSPC conditioned medium to be less than 50% of those in SGZ-NSPC counterpart (Fig. 6f, g). IGF2 secretion into SGZ-NSPC culture medium was significantly induced by OGD but reduced by RBM3 depletion (Fig. 6g). Conversely, RBM3 expression had no effect on IGF2 release into SVZ-NSPC culture medium (Fig. 6f). Overall, hypothermia inhibited IGF2 secretion in both SVZ- and SGZ-NSPCs after OGD, probably by reducing its expression level (Fig. 6f, g).

**Fig. 7:**
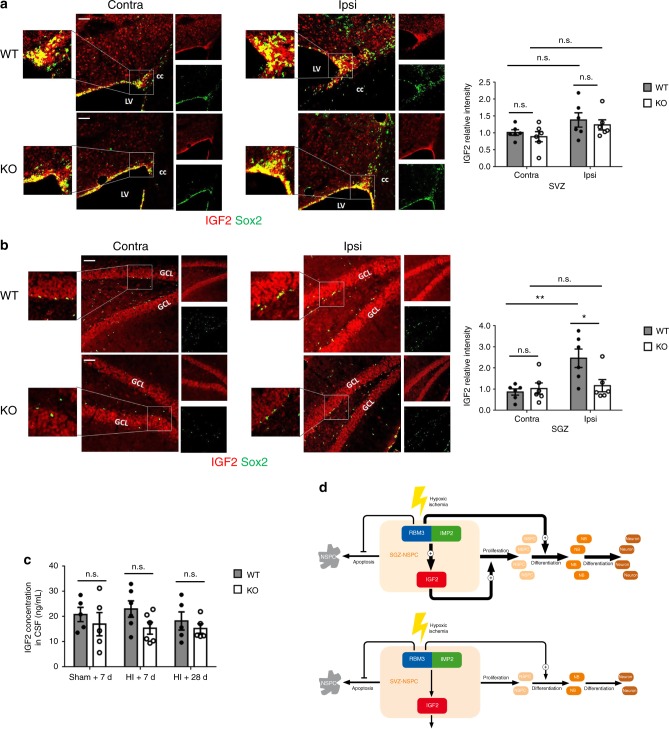
RBM3 regulates IGF2 expression in SGZ but not in SVZ in vivo. **a**, **b** Representative immunofluorescent IGF2 staining in SVZ (**a**) or SGZ (**b**) of RBM3 WT and KO animals treated with HI and recovered for 7 days. All images were captured with the same parameters. Sox2+ NSPCs were co-stained to identify the SVZ or SGZ. Relative IGF2 intensity in indicated area was quantified with Image J. Six animals were counted per group (*n* = 6). Scale bar: 50 µm. LV lateral ventricle, cc corpus callosum, GCL granular cell layer, Contra contralateral (uninjured side), ipsi ipsilateral (injured side). Repeated measures two-way ANOVA was used for statistical analysis; n.s. not significant; *p < 0.05; **p < 0.01. **c** ELISA measurement of IGF2 levels in cerebrospinal fluid (CSF). CSF samples were collected from WT or KO mice treated with sham operation, HI plus 7 days recovery or HI plus 28 days recovery (Sham+7d: *n* = 5, HI + 7d: *n* = 6, HI + 28d: *n* = 5). Two-way ANOVA was used for statistical analysis; n.s. not significant. All data are presented as mean ± SEM. **d** Hypothesized model. In SGZ-NSPC, RBM3 interacts with IMP2 and thereby enhances IGF2 expression and release to promote SGZ-NSPC proliferation in an autocrine pattern after HI injury. On the other hand RBM3 promotes neuronal differentiation. In SVZ-NSPC, RBM3 does not promote proliferation after HI injury and affects neuronal differentiation to a lesser extent. In both cell types, RBM3 inhibits apoptosis. NB neuroblast

Additionally, we noticed that the intensity of IGF2 protein expression was enhanced in the ipsilateral SGZ but not SVZ in WT mice 7 days after HI injury (Fig. 7a, b). In RBM3-deficient mice, the intensity of IGF2 expression was reduced in the ipsilateral SGZ compared to WT, while remaining unchanged in the ipsilateral SVZ (Fig. 7a, b), in accordance with in vitro findings. Finally, we measured IGF2 levels in cerebrospinal fluid (CSF) by enzyme-linked immunosorbent assay (ELISA) in three groups: sham operation plus 7 days recovery, HI injury plus 7 days recovery and HI injury plus 28 days recovery. No significant change in CSF IGF2 levels was observed between WT and KO in all three groups (Fig. 7c), indicating that RBM3 does not alter IGF2 secretion from the epithelial cells of the choroid plexus, considered the main source of CSF^24^. We further found that RBM3 expression was absent in choroid plexus, while IGF2 was high as reported previously^24^ (Supplementary Fig. 7h). This finding supports our notion that RBM3 does not regulate IGF2 level in CSF thus does not affect the proliferation of SVZ-NSPC.

To sum up, RBM3 interacts with IMP2 in NSPCs; however, only in SGZ-NSPC does RBM3 facilitate IMP2-mediated stabilization of *IGF2* mRNA and promote IGF2 expression and secretion after HI injury.

## Discussion

While injured neurons lose synaptic connectivity and undergo cell death after HI, counteracting endogenous regenerative processes are activated, leading to neurogenesis and synaptogenesis^29^. Therapeutic hypothermia is widely known to protect the brain from HI injury^30,31^, but there is limited and inconsistent information as to whether hypothermia promotes or inhibits injury-induced NSPC proliferation and neuronal differentiation or if anti-apoptosis is the key mechanism^16^. Here we demonstrate that under physiological conditions, RBM3 is expressed in brain, yet its deficiency has no obvious effect on brain development in vivo, probably due to other unknown compensating mechanisms. However, under pathological conditions such as HI, RBM3 is indispensable for neuroprotection and post-injury neuroregeneration. RBM3 not only protects NSPCs from HI-induced apoptosis, it also stimulates NSPC proliferation and neuronal differentiation. We demonstrate that RBM3 promotes NSPC proliferation after HI by regulating the IMP2-IGF2 pathway in SGZ-NSPCs but not SVZ-NSPCs (Fig. 7d).

Cold inducible RNA-binding protein (CIRP) is the only known homologous protein of RBM3 in mammals^6^. Like RBM3, it has been reported to promote the proliferation and suppress the apoptosis of neural stem cells in vitro^32,33^. However, CIRP has been identified as a damage-associated molecular pattern (DAMP) molecule, inducing detrimental inflammatory responses^34^. Extracellular CIRP levels increase in ischemic stroke models causing massive neuronal damage^35^. In contrast, no such deleterious effect has been reported with RBM3, which is therefore considered a safer modulator of post-injury neurogenesis than CIRP.

IMP family proteins are widely expressed and play important roles at different stages of development. In adulthood, in contrast to IMP1 and IMP3, only IMP2 remains relatively highly expressed, playing a major role in cell survival^28,36^, NSPC proliferation^37^, and neuronal differentiation^27^. As the main downstream factor of IMP2, IGF2 is abundant in the proliferative regions of both embryonic and adult brain^22^, similarly to the temporal and spatial expression pattern of IMP2 and RBM3. In recent years, IGF2 has been identified as a positive regulator of the proliferation of embryonic and neonatal NSPCs^38,39^ and adult NSPCs^23,24^. Interestingly, SVZ-NSPC responses to exogenous IGF2 follow a paracrine pattern, while SGZ-NSPCs secrete IGF2 and regulate self-renewal in an autocrine manner^23,24^. Our results support the hypothesis that RBM3 upregulates IGF2 expression and secretion in SGZ-NSPCs but not SVZ-NSPCs, explaining why only SGZ-NSPC proliferation is affected by RBM3 expression level after HI injury. Like RBM3, microprocessor complex subunit DGCR8, a key protein involved in miRNA biogenesis, can also regulate IGF2 expression and promote the proliferation of SGZ-NSPCs but not SVZ-NSPCs^40^. As IGF2 has been proved to consolidate and enhance memory, a major hippocampal function^41^, a decrease in IGF2 induced by RBM3 deficiency can be expected to affect memory function after HI injury, and may also contribute to discovered memory loss by RBM3 silencing in a chronic neurodegenerative disease model^7^.

It is feasible to design a small compound to stabilize the RBM3 protein and maintain its expression at high levels by targeting its typical RNA-recognition motif, in order to promote endogenous neurogenesis, particularly in the hippocampus. Such a compound could serve to treat acute brain injury and chronic disease. A preliminary successful example has already been developed with the CIRP homologue, although its activity requires further tests^42^. In addition, the safety of RBM3-based therapy will need to be carefully evaluated for potential tumorigenesis. As IMP family members and IGF2 are critical in promoting cell proliferation, they maintain stem cell stemness under physiological conditions, but are also thought to favor cancer cell progression in diverse tumor types along with their high expression^28,36^. Fortunately, clinical studies have revealed that in contrast to its CIRP homologue, high RBM3 expression is associated with favorable outcome in various cancers^6^. Although the underlying mechanisms remain largely unknown, this suggests that RBM3-targeted therapy could be safe to administer in brain disorders.

## Methods

### Mice

All animal experiments were approved by the veterinary office of Basel city (authorization number 2064 and 2652) and were in accordance with the guidelines on laboratory animals. RBM3 knockout (KO) C57BL/6 mice were kindly provided by Prof. Tadatsugu Taniguchi (University of Tokyo, Japan). As RBM3 is X-chromosome-linked gene, only male RBM3 WT or KO mice were used in this study. All the mice were maintained under standard conditions of 12/12 h of light/dark at 25 °C before and after surgery.

### Hypoxic-ischemia (HI) model

From 2- to 3-months-old adult mice were anesthetized by 3% isoflurane. Right common carotid artery (RCCA) was exposed and permanently ligated by electrocauterization and subsequently cut. RCCA was only exposed but not ligated in sham animals. After recovery from surgery, animals were subjected to 8% hypoxia for 20 min at 37 °C. Sham animals were not treated with hypoxia. After hypoxic stress, all animals were intraperitoneally injected with 50 mg/kg BrdU. Subsequent BrdU injection was performed every other day for 7 days. Mice were sacrificed 7 days or 28 days after HI injury for further analysis.

### Immunofluorescent staining

Mice were perfused transcardially with 4% paraformaldehyde (PFA). Brains were collected and post-fixed with 4% PFA for 24 h and immersed in 30% sucrose for additional 24 h both at 4 °C, then embedded in O.C.T. (TissueTek) and frozen in isopentane. Cryoprotected brains were cut into 25 µm thick coronal sections in a cryostat (Leica). For cultured NSPCs, cells were seeded onto poly-L-lysine coated 16-well chamber slide (Nunc LabTek) and fixed with 4% PFA for 10 min at RT. To stain BrdU, brain sections or cells were treated with 2 M HCl at RT for 1 h and neutralized with 0.1 M sodium borate (pH 8.5) for 10 min before blocking. 0.5% Trition X-100 and 5% normal goat serum in phosphate buffer was used for permeabilization and blocking. Samples were incubated with primary antibodies overnight at 4 °C. Alexa Fluro dye conjugated secondary antibodies were used in 1:500 (Thermo Fisher). Nuclei were couterstained with DAPI. Information for primary antibodies was listed in Supplementary Data 4.

### Cresyl violet staining

Brain cryosections were mounted on Superfrost PLUS glass slides and stained with 0.2% cresyl violet solution for 20 min at RT. Stained slides were subsequently washed in distilled water, dehydrated in 70, 80, 90, 95, and 100% ethanol, and then cleared twice in xylene. Eukitt mounting medium (Fluka) was used for mounting slides.

### Stereology, imaging, and cell quantification

Stereological coordinates were identified according to adult mouse brain atlas^43^. Animals 7 days after HI injury were used for infarction volume estimation. The infarction volume estimation method was adapted from elsewhere using the Cavalieri estimator probe^44^. Fixed brains were serially cut into 25 µm thick coronal sections in a cryostat (approximately between +2.0 and −4.0 mm from the bregma), and every twelfth sections were picked (300 µm interval) for cresyl violet staining as described above, totally 20 sections. Imaging was performed in live mode with a ×5 objective using an Axio Imager Z1 microscope (Zeiss) equipped with Stereo Investigator (MBF Bioscience). The contours of direct infarction areas (mm^2^), ipsilateral hemisphere areas (mm^2^) and contralateral hemisphere areas (mm^2^) were outlined and calculated by Cavalieri point-counting estimator from Stereo Investigator. A grid spacing of 50 µm was used. The corrected infarction areas were calculated as follows:

1 $$A_{{\mathrm{corrected}}} = A_{{\mathrm{direct}}} - \left( {A_{{\mathrm{ipsi}}} - A_{{\mathrm{contra}}}} \right)$$

*A*_*corrected*_ is the corrected infarction area, *A*_*direct*_ is the direct infarction area, *A*_*ipsi*_ is ipsilateral hemisphere area, and *A*_*contra*_ is contralateral hemisphere area. The estimated infarction volume (mm^3^) was calculated by summing up corrected infarction areas from all 20 sections and then multiplying by the 300 µm interval. The SVZ (tail and lateral wall) or DG (granular cell layer and hilus) volumes (mm^3^) were estimated in a direct way without correction, using Cavalieri point-counting estimator as described above, but with 20 µm grid spacing. Four sections containing the SVZ (approximately between +1.2 and 0.0 mm from the bregma) and eight sections containing the entire DG (approximately between −1.2 and −3.6 mm from the bregma) per animal were used for volume estimation.

Cell quantification in the SVZ (approximately between +1.2 and 0.0 mm from the bregma) were estimated with every twelfth serial coronal sections (25 µm thick each, 300 µm interval), totally four sections. Cell quantification in the SGZ covering dorsal and ventral hippocampus (approximately between −1.2 and −3.6 mm from the bregma) were estimated with every twelfth serial coronal sections (25 µm thick each, 300 µm interval), totally eight sections. Double-positive cells were imaged using z-stack function with LSM710 confocal microscope (Zeiss). Stacked images were acquired every 1 µm throughout the section (25 optical sections) and presented in orthogonal view to confirm co-localization. Cell counting in the SVZ or DG was performed with Optical Fractionator from Stereo Investigator (MBF Bioscience) according to published methods^14,45–47^. The counting frame size of 30 × 30 µm was used for all the counting types. To count total BrdU+, BrdU+/Sox2+, BrdU+/Dcx+ cells in the SVZ and DG, or TUNEL + cells in the SVZ in HI + 7d groups, a 60 × 60 µm sampling grid was used. For TUNEL + cells in the DG in HI + 7d groups, a 90 × 90 µm sampling grid was used. For total BrdU + and BrdU + NeuN + cells in the SVZ and DG in HI + 28d groups, a 30 × 30 µm sampling grid was used. Cells were only counted in the optical disector with the height of 10 µm, but not counted in the top and bottom 3 µm guard zones. Total cell number (N) was estimated with the following calculation formula:

2 $$N = {\sum} {Q^ - \times \left( {t/h} \right) \times \left( {1/asf} \right) \times \left( {1/ssf} \right)}$$

*∑Q*^−^ is total cell count in disector; *t* is section thickness after processing; *h* is optical disector height; *asf* is area sampling fraction (counting frame size/sampling grid size); and *ssf* is slice sampling fraction (1/section interval). The density of positive cells in the two neurogenic niches was determined by dividing the total positive cell number to the SVZ or DG volume, respectively. The SVZ or DG volumes (mm^3^) were estimated on adjacent section series with the method as described above. For non-operating and sham animals, the mean of left and right hemisphere cell numbers was presented for each animal.

For relative intensity quantification of IGF2, all the images were captured with the same parameters. The signal in lateral ventricle was subtracted as background. The average intensity of IGF2 signal in the SVZ or SGZ from all sections prepared as above mentioned was calculated with Image J (National Institutes of Health) and was presented for each animal.

For cultured cells, images of five random fields with ×20(except ×40 for proximity ligation assay) objective lens were captured with AX70 fluorescent microscope (Olympus) for quantification per experiment. Three independent experiments were performed.

### Cell culture

HEK293 cells were cultured in DMEM (Gibco) medium supplemented with 10% FBS (Gibco). SVZ-NSPCs and SGZ-NSPCs were isolated from the SVZ of lateral ventricle or DG of 2–3-months-old adult male mice respectively, according to previous protocols^48,49^. The whole brain excluding cerebellum and meninges from postnatal day 0 male mice was used for NSPC culture. In brief, cells from desired regions were dissociated by papain (Worthington) and DNase I (Sigma) digestion, and passed through 40 µm cell strainer (Sigma) to remove cell clusters. Isolated NSPCs were cultured in complete DMEM-F12 medium (Gibco) supplemented with 1 × B27 supplement (Gibco), 2 mM L-glutamine (Gibco), 20 ng/mL EGF (PeproTech) and 20 ng/mL FGF2 (PeproTech). Cells were maintained as neurospheres in uncoated dishes or as monolayer in poly-L-lysine (Sigma) coated dishes or 16-well chamber slides (LabTek). To passage neurospheres, 0.25% Typsin (Sigma) was used to digest neurospheres into single cells.

### Plasmids and constructs

pCEP4-RBM3 construct used to overexpress RBM3 in primary NSPCs and control empty pCEP4 vector (Thermo Fischer) were described in previous report^9^. Commercial pCMV3-RBM3 was intended for mammalian overexpression of full-length RBM3 cDNA under CMV promoter with additional N-terminal HA tag (Sino Biological, HG16437-NY). Commercial pCMV3-IMP2 plasmid was designed for full-length IMP2 overexpression with N-terminal FLAG tag (Sino Biological, HG11116-NF). Control empty vectors with N-terminal HA or FLAG tag were purchased from Sino Biological as well (pCMV3-N-HA-NCV and pCMV3-N-FLAG-NCV).

RRM and KH domains of IMP2 including two RRMs and four KHs respectively, were cloned into pCMV3-N-FLAG-NCV with FLAG tag. IMP2-RRMs were subcloned by standard PCR using IMP2-RRMs F/R primer pairs. IMP2-KHs were amplified by overlapping PCR to conjugate DNA encoding affinity tag to DNA encoding truncated peptides. pCMV3 F/FLAG-tag R and IMP2-KHs F/R primer pairs were used for overlapping PCR. Overlapped PCR products were digested with KpnI/NotI and ligated to digested pCMV3-N-FLAG-NCV vector. Primers sequences were listed in Supplementary Data 4. All the pCMV3 constructs were transiently expressed in HEK293 cells.

### Transient transfections

For co-immunoprecipitation, all pCMV3-based plasmids expressing full-length or truncated RBM3 or IMP2 were transiently transfected into HEK293 cells with FuGENE HD Transfection Reagent (Promega) for 48 h. One to two micrograms of each plasmid was used to transfect 1 × 10^6^ HEK293 cells. For cultured NSPCs, 10 µg pCEP4 empty vector or pCEP4-RBM3 plasmid were transiently transfected into 5 × 10^6^ NSPCs by electroporation, using Mouse Neural Stem Cell Nucleofector Kit (Lonza) and program A-33 of Nucleofector I Device (Lonza). Transfected cells were kept in 12-well plate overnight and then spun down at 100 x g at RT for 5 min to remove dead cells. The remaining viable cells were counted and seeded at the density of 1 × 10^4^ cells/mL into poly-L-lysine coated 16-well chamber slide. Cells were incubated for another 48 h before starting further treatments.

### Oxygen-glucose deprivation (OGD)

NSPCs were cultured in poly-L-lysine coated 16-well chamber slide or petri dish with diameter of 10 cm for 24 h. To introduce OGD, normal NSPC culture medium was changed to glucose-free complete medium (using glucose-free DMEM-F12, Biowest) and slides or dishes were kept in hypoxic chamber (Elektrotek) with nitrogen flush for 15 min. Then the chamber was sealed and kept at 37 °C for 3 h. After OGD, glucose-free medium was switched back to normal complete culture medium and cells were incubated in normal conditions with 20% oxygen at 37 °C (normothermic group) or 32 °C (hypothermic group) for indicated period. For BrdU incorporation, 20 μM BrdU was added into the culture medium immediately after OGD or mock treatment.

### Quantitative RT-PCR

Total RNA from cells or brain tissues were isolated by ReliaPrep RNA Miniprep System (Promega). One microgram total RNA was reversely transcribed into complementary DNA using GoScript Reverse Transcription System (Promega). Quantitative amplification was carried out with GoTaq qPCR Master Mix (Promega) in 15 μL reaction volume. Thermal cycles were performed on CFX Connect Real-Time PCR Detection System (Bio-Rad) at 95 °C for 5 min, followed by 40 cycles of 95 °C for 15 s and 60 °C for 1 min. 2^*−*ΔΔCT^ method was used to calculate the fold change of gene expression. Three independent experiments were performed. Primers sequences were listed in Supplementary Data 4.

### Western blot

Cells or brain tissues were harvested, washed with cold PBS and lysed in lysis buffer (1% Triton X-100, 50 mM Tris, 150 mM NaCl, 1 × Roche Protease Inhibitor Cocktail, pH 8.0). Brain tissues were further homogenized with Dounce tissue grinder on ice. After centrifugation at 15000 rpm for 10 min at 4 °C, supernatants were collected and normalized with RC DC Protein Assay (Bio-Rad). Lysates were loaded onto NuPAGE Novex 4–12% Bis-Tris protein gels (Invitrogen) and transferred to PVDF membranes (Amersham/GE Healthcare Life Sciences). Samples were incubated with primary antibodies overnight at 4 °C. HRP-linked anti-rabbit IgG and HRP-linked anti-mouse IgG secondary antibodies were purchased from Cell Signaling Technology and used in 1:5000. Information for primary antibodies was listed in Supplementary Data 4.

### Neurosphere assay

Neurospheres assay was performed to test self-renewal capacity of cultured NSPCs. Pre-cultured NSPCs directly from dissociated tissue in the form of neurospheres were digested into single cells by trypsin and plated at the density of 100 cells per 100 µL medium in each well of 96-well plate to form primary neurospheres. Primary neurospheres were further digested into single cells and plated at the same density to form secondary neurospheres. After 48 h culture, the numbers of primary and secondary neurospheres were counted in two groups in terms of their diameters (<20 and ≥20 µm). Triplicates were included in each group.

### Differentiation assay

NSPCs were seeded in 16-well chamber slide as monolayer at the density of 1 × 10^5^/mL and maintained for 24 h at 37 °C. Cultured cells were subjected to directed differentiation or OGD-induced differentiation. For directed differentiation, NSPCs were incubated in the following medium for 7 days before immunostaining: Neuralbasal medium supplemented with 1 × B27 supplement and 2 mM L-glutamine for neuronal differentiation; DMEM supplemented with 1 × N-2 supplement (Gibco), 2 mM L-glutamine and 1% FBS for astrocyte differentiation; Neuralbasal medium supplemented with 1 × B27 supplement, 2 mM L-glutamine and 30 ng/mL triiodothyronine (T3) solution (Calbiochem) for oligodendrocyte differentiation. For OGD-induced differentiation, cells were challenged with 3 h OGD and then reoxygenated at 37 °C for 2 days in NSPC complete culture medium, followed by 5 days in above-mentioned directed differentiation medium, respectively. The following primary antibodies were used to identify various differentiated cells: MAP2 for neurons; Dcx for neuroblasts; S100 for glia cell progenitors and Olig2 for oligodendrocyte progenitor cells (OPCs). Information for primary antibodies was listed in Supplementary Data 4. All quantifications were performed in triplicates.

### TUNEL staining

TUNEL staining was used to test late apoptosis. The assay was performed with Click-iT Plus TUNEL Assay for In Situ Apoptosis Detection with Alexa Fluor 488 Dye (Invitrogen) following manufacturer’s instructions. Brain cyrosections were digested with Protease K for antigen retrieval and counterstained with DAPI. Cultured NSPCs were permeabilized with Triton X-100 and counterstained with DAPI. TUNEL+ cells were counted in brain sections or cultured NSPCs, respectively. In cultured NSPCs, TUNEL+ cell percentages in all DAPI stained cells were calculated in triplicates.

### Co-immunoprecipitation and RNA-immunoprecipitation

For co-immunoprecipitation (CoIP), 4 μg primary antibodies or control normal IgG were conjugated to 40 μL Dynabeads Protein G (Invitrogen) for 45 min at RT. HEK293 cells which were transfected with full-length or truncated RBM3 and IMP2 overexpressing pCMV plasmids (as described above) were harvested 48 h after transfection. HEK293 or P0 NSPC cell lysates were incubated with antibody-coupled Dynabeads Protein G overnight at 4 °C. For RNase-treated group, cell lysates were pretreated with 10 U/μl RNaseT1 (Fermentas) for 15 min at RT before subjected to beads. Proteins were eluted from beads in NuPAGE LDS Sampler Buffer (Invitrogen) containing 50 mM DTT at 70 °C for 10 min. Samples were analyzed by Western blot. RNA-immunoprecipitation (RIP) was performed in a native way similar to CoIP. After mock or OGD treatment, the starting material was adjusted to 5 × 10^6^ cells per sample. All the reagents contained 40 U/mL RNase inhibitor RNasin (Promega) and prepared in RNase-free water (Promega) to minimize the activity of RNase from the environment. Immunoprecipiated RNA was eluted in lysis buffer from above-mentioned total RNA isolation kit at 70 °C for 10 min, and further purified by the kit. Samples were analyzed by quantitative RT-PCR.

### Proximity ligation assay

Proximity ligation assay (PLA) was used to reveal protein interactions between RBM3 and IMP2 in situ using Duolink In Situ Red Starter Kit Mouse/Rabbit (Sigma). Brain sections and cultured NSPCs were counterstained with DAPI. In cultured NSPCs, PLA + dots (each dot represents one interaction) in each cell were quantified, and 25 cells per sample were analyzed for statistical analysis.

### RNA sequencing

Hippocampi were isolated from postnatal day 3 (P3) or adult RBM3 WT or KO mice (*n* = 3 for each group) and homogenated in Trizol by Dounce tissue grinder on ice. Total RNA was separated by chloroform and precipitated by isopropanol, and then further cleaned up using RNA Clean & Concentrator Kit (Zymo Research). Complementary DNA libraries were constructed from messenger RNA and qualified on Agilent 2100 Bioanalyzer. RNA sequencing was carried out on Illumina Hiseq-2500 sequencing system with single reads of 50 bp read length. Differentially expressed genes (DEGs) were filtered with log2A > 3.32 (Average counts per million (CPM) > 10); |log2 FC| > 0.26 (|Fold change| > 1.2) and unadjusted P value < 0.05. Venn diagrams were generated via InteractiVenn online tool (http://www.interactivenn.net). Gene heatmap were generated via Heatmapper online tool (http://heatmapper.ca/expression). Gene set enrichment analysis (GSEA) was performed via online analysis tool (http://www.webgestalt.org). Volcano plot and M-A plot were prepared by GraphPad Prism 8.0. The raw data are available on NCBI BioProject (Project ID: PRJNA529585).

### ELISA

Cerebrospinal fluid (CSF) samples were collected from cisterna magna immediately before sacrificing animals as described previously^50^. NSPC culture media were collected immediately before fixing cells. IGF2 levels in CSF or in culture medium were measured by mouse IGF2 ELISA Kit (Abnova) with the sensitivity of <5 pg/mL. Five replicates for sham and HI + 28d groups and six replicates for HI + 7d group were included. Culture medium from NSPCs were in five replicates.

### Statistical analysis

All in vitro experiments were repeated at least three times. All in vivo experiments included a minimum of five mice per group. Quantification data were presented in standard error mean (SEM). For comparison of two groups, statistical significance was determined by two-tailed unpaired *t*-test with or without Welch’s correction (single factor), two-way ANOVA (two factors) followed by Tukey’s multiple comparison test or Sidak’s multiple comparison test, and three-ANOVA (three factors) followed by Sidak’s multiple comparison test. For the quantification of data involving contralateral and ipsilateral sides from the same animal, repeated measures two-way ANOVA was performed, and mixed effects model was applied for further comparison with the sham group. p value less than 0.05 was considered significant. n.s. not significant; *p < 0.05; **p < 0.01; ***p < 0.001; ****p < 0.0001. Statistical analysis was performed using GraphPad Prism 8.0 and all details are reported in Source Data file.

### Reporting summary

Further information on research design is available in the Nature Research Reporting Summary linked to this article.

## Supplementary information

Supplementary Information

Peer Review

Reporting Summary

Description of Additional Supplementary Files

Supplementary Dataset 1

Supplementary Dataset 2

Supplementary Dataset 3

Supplementary Dataset 4

Source Data

## Data Availability

The raw RNA-seq data are available on NCBI BioProject (Project ID: PRJNA529585). The source data relating to Figs. 1b, c, e–h, 2b–e, 3b–i, 4b, c, e–h, 5b, 6a–d, f, g, 7a–c and Supplementary Figs. 1a, b, e–h, 2a–f, 5c–f, h–k, 7a are provided in the Source Data file. Uncropped blots are shown in Supplementary Fig. 8. All other raw data are available from the authors upon request. A reporting summary for this article is available as a Supplementary Information file.
