# Supplementary material for: RBM3 promotes neurogenesis in a niche-dependent manner via IMP2-IGF2 signaling pathway after hypoxic-ischemic brain injury
Source: Nat Commun. 2019 Sep 4;10:3983. doi: 10.1038/s41467-019-11870-x (PMC6726629; doi:10.1038/s41467-019-11870-x)
Supplement: Supplementary file 1 — Supplementary Information [file 41467_2019_11870_MOESM1_ESM.pdf]

Supplementary Information

RBM3 promotes neurogenesis in a niche-dependent manner via IMP2-IGF2 signaling pathway after hypoxic-ischemic brain injury

Zhu *et al.*

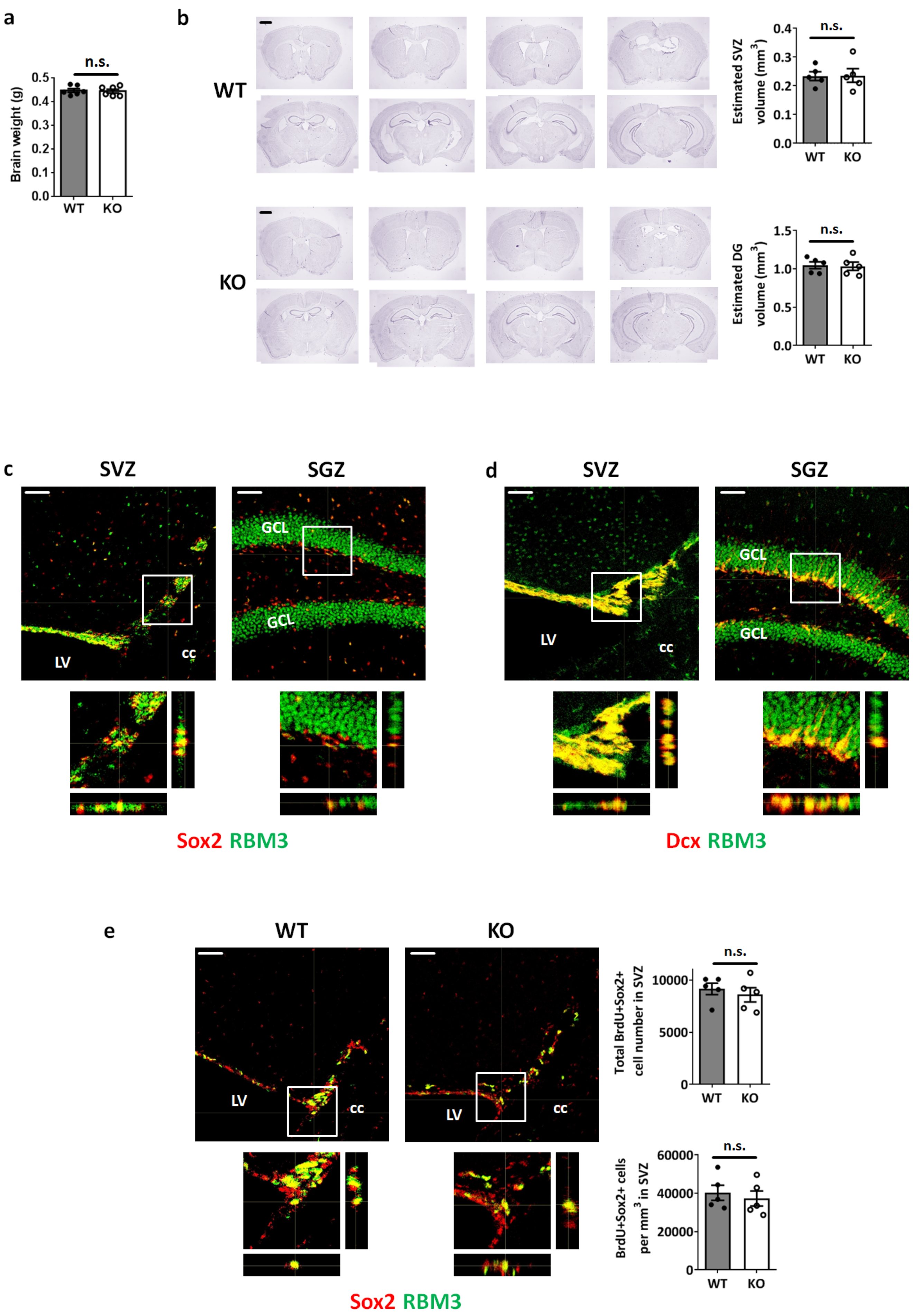

Supplementary Figure 1 (to be continued)

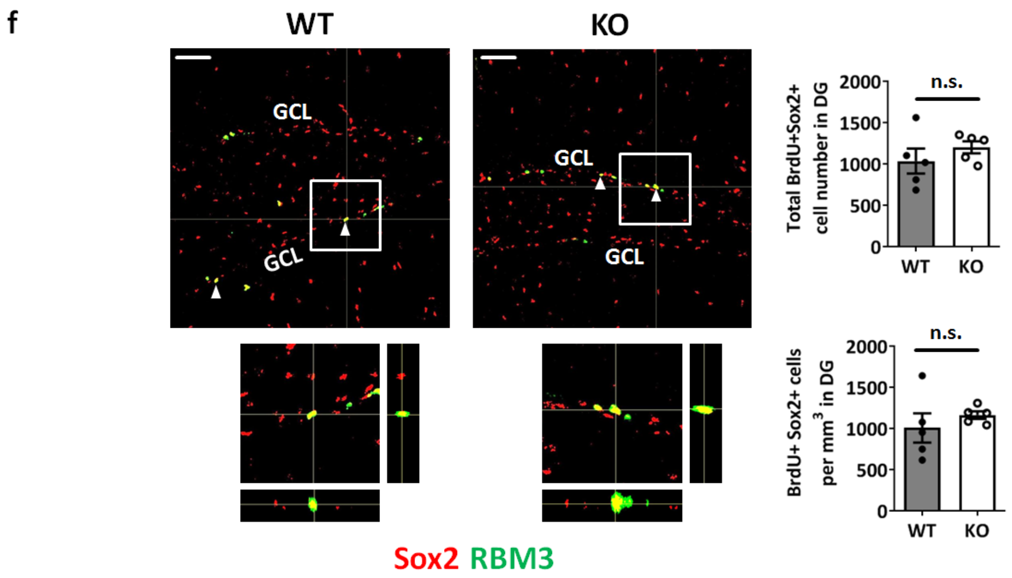

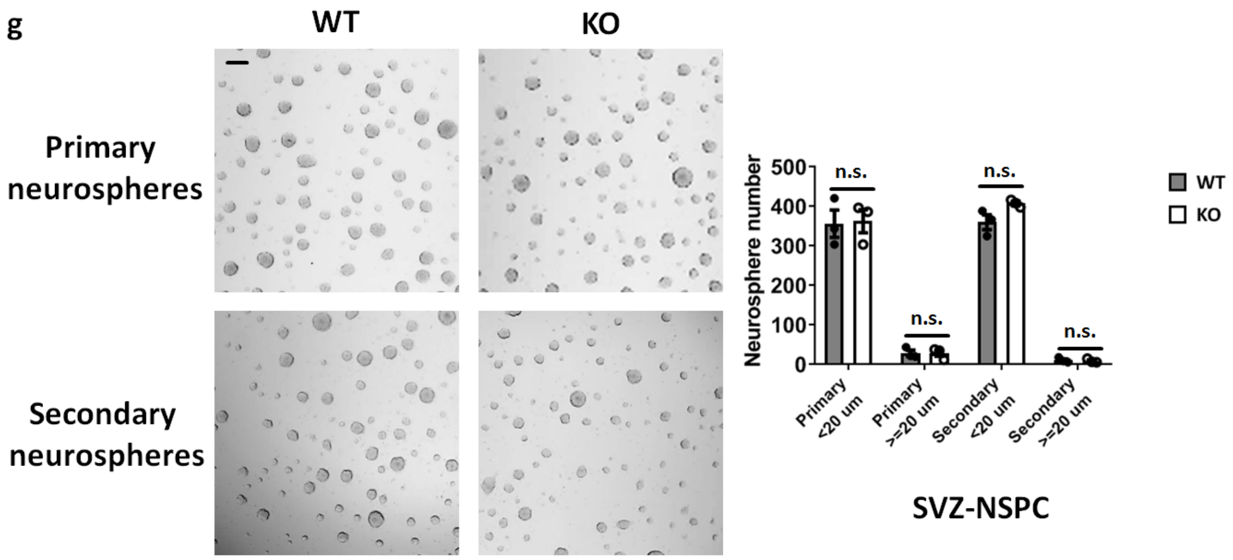

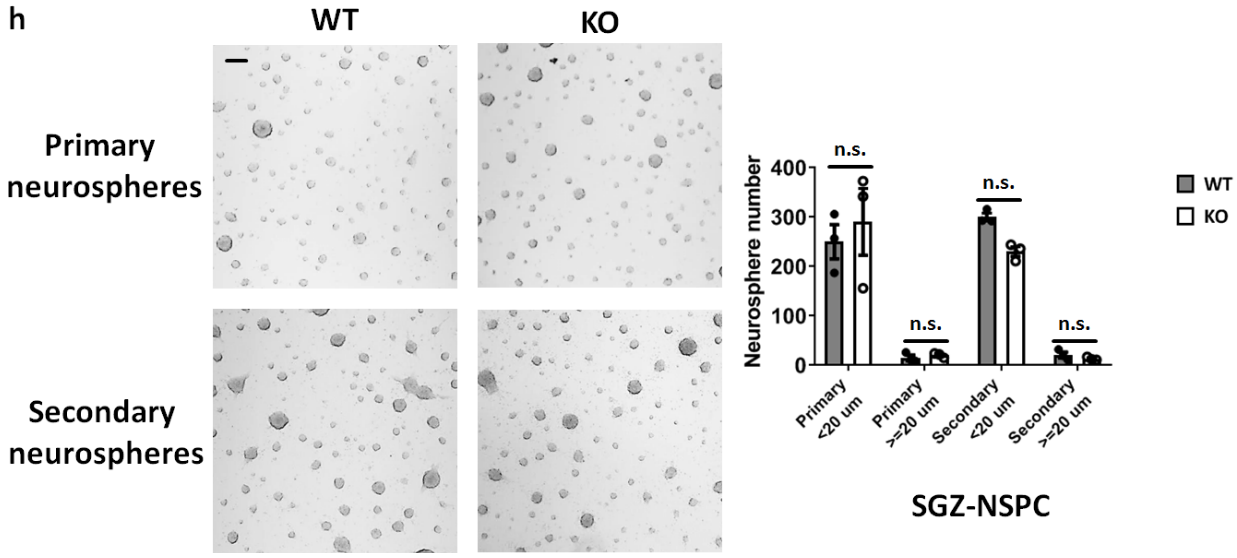

Supplementary Figure 1 (continued)

Supplementary Figure 1 RBM3 KO mouse brain does not show evident abnormality in physiological conditions.

a. Brains from adult RBM3 WT or KO mice were collected and measured (six animals per group, n=6), and analyzed by two-tailed t-test. n.s., not significant. **b.** Representative cresyl violet staining of adult RBM3 WT or KO brain without injury. Scale bar: 1 mm. The volume of SVZ and DG were estimated as described in methods, and compared between WT and KO. Five animals were counted per group (n=5). Two-tailed t-test was used for statistical analysis; n.s., not significant. **c-d.** Representative immunofluorescent staining of RBM3 and Sox2 **(c)**, or RBM3 and Dcx **(d)** in the SVZ and DG of adult WT mouse brain. Orthogonal view confirmed the co-localizations. Scale bar: 50 µm. LV: lateral ventricle; cc: corpus callosum; GCL: granular cell layer. **e-f.** RBM3 WT or KO mice were intraperitoneally injected with BrdU for 7 days to monitor NSPC proliferation. Representative immunofluorescent staining images of BrdU and Sox2 in the SVZ **(e)** or DG **(f)** were demonstrated (five animals per group, n=5). Orthogonal view confirmed the co-localization of BrdU and Sox2. Scale bar: 50 µm. LV: lateral ventricle; cc: corpus callosum; GCL: granular cell layer. Two tailed t-test was used for statistical analysis. n.s. not significant. **g-h.** SVZ-NSPCs and SGZ-NSPCs were cultured *in vitro* and seeded into 96-well plates in equal amounts for the analysis of primary neurospheres after pre-culture or passaged for analyzing secondary neurospheres in SVZ-NSPC **(g)** or SGZ-NSPC **(h)**. Neurospheres with diameters <20 µm or >=20 µm were counted and analyzed separately. Three independent experiments were performed (n=3). Scale bar: 50 µm. Three-way ANOVA was used for statistical analysis. n.s. not significant. All data are presented in SEM.

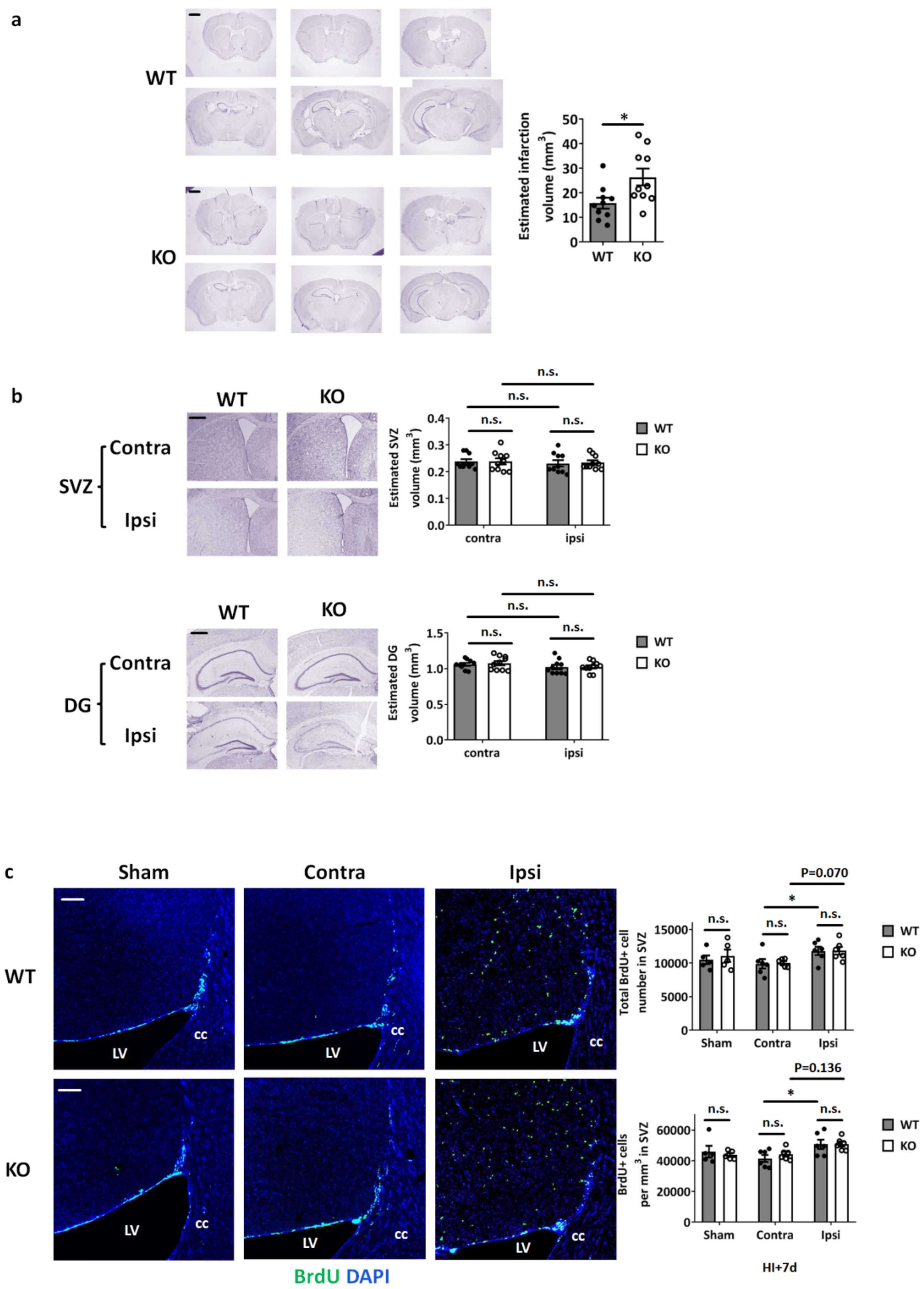

Supplementary Figure 2 (to be continued)

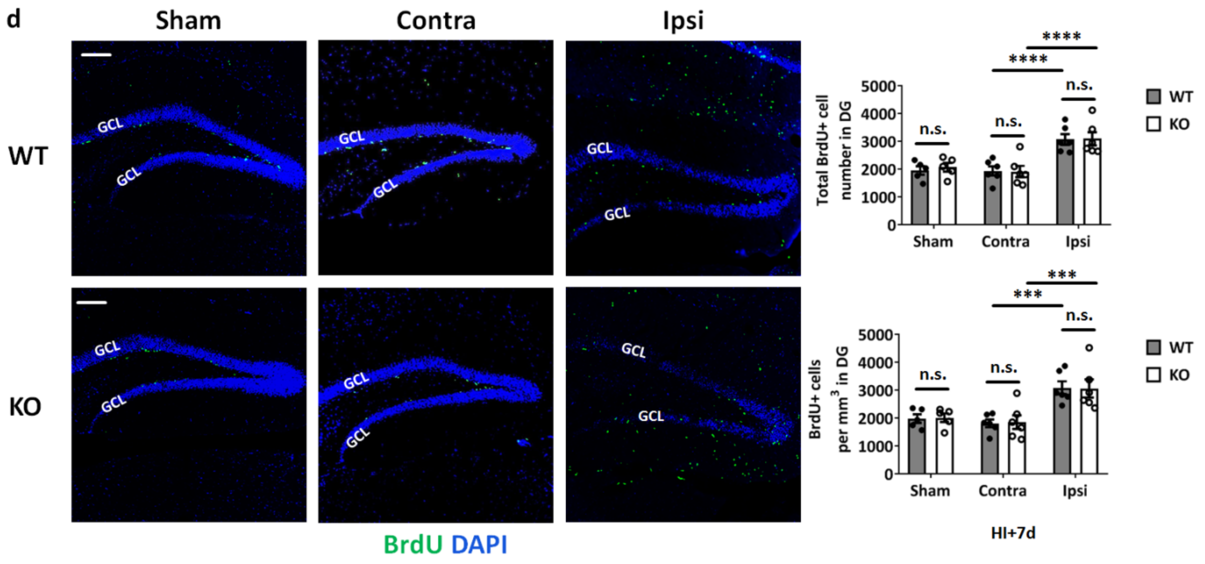

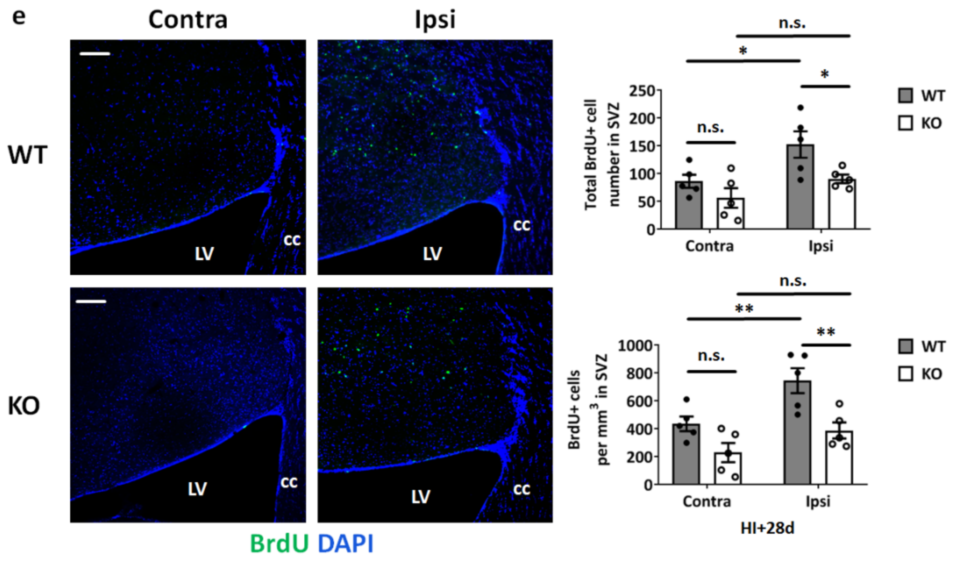

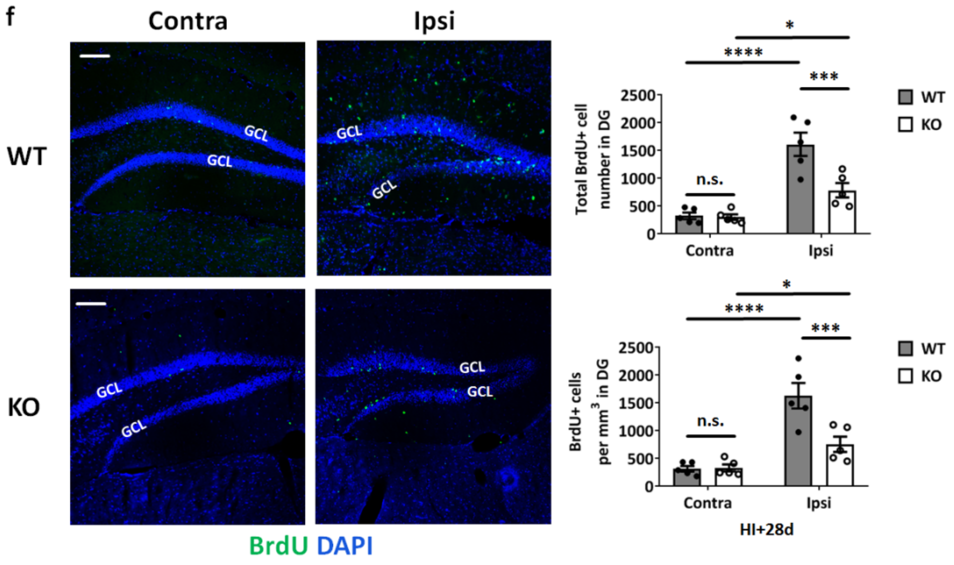

Supplementary Figure 2 (continued)

Supplementary Figure 2 HI injury caused tissue damage and activated cell proliferation in both RBM3 WT and KO mice.

a. Representative cresyl violet staining of adult RBM3 WT or KO brains after HI injury plus 7 days recovery. Scale bar: 1 mm. The infarction volumes were estimated as described in Methods. Two-tailed t-test was used for statistical analysis (ten animals per group, n=10); * p<0.05. **b.** High magnification of the SVZ and DG regions from **(a)**. Scale bar: 500 µm. The SVZ volumes and DG volumes were calculated as described in Methods. Repeated measures two-way ANOVA was used for statistical analysis (ten animals per group, n=10); n.s. not significant. **c-d.** Representative BrdU and DAPI staining in SVZ **(c)** and DG **(d)** of RBM3 WT and KO animals treated with HI and recovered for 7 days with BrdU injection every other day. Animals in control group received sham surgery. Total BrdU+ cell number and density in the SVZ and DG were estimated. Five sham animals and six HI animals were counted per group (Sham: n=5, HI: n=6). Sham: sham group; contra: contralateral (uninjured side) in HI group; ipsi: ipsilateral (injured side) in HI group. Scale bar: 100 µm. LV: lateral ventricle; cc: corpus callosum; GCL: granular cell layer. Repeated measures two-way ANOVA was used for statistical analysis; n.s. not significant; * p<0.05; *** p<0.001; **** p<0.0001. **e-f.** Representative BrdU and DAPI staining in SVZ **(e)** and DG **(f)** of RBM3 WT and KO animals treated with HI and recovered for 28 days with BrdU injection every other day in the first 7 days. Total BrdU+ cell number and density in the SVZ and DG were estimated. Five animals were counted per group (n=5). Contra: contralateral (uninjured side); ipsi: ipsilateral (injured side). Scale bar: 100 µm. LV: lateral ventricle; cc: corpus callosum; GCL: granular cell layer. Repeated measures two-way ANOVA was used for statistical analysis; n.s. not significant; * p<0.05; ** p<0.01; *** p<0.001; **** p<0.0001. All data are presented in SEM.

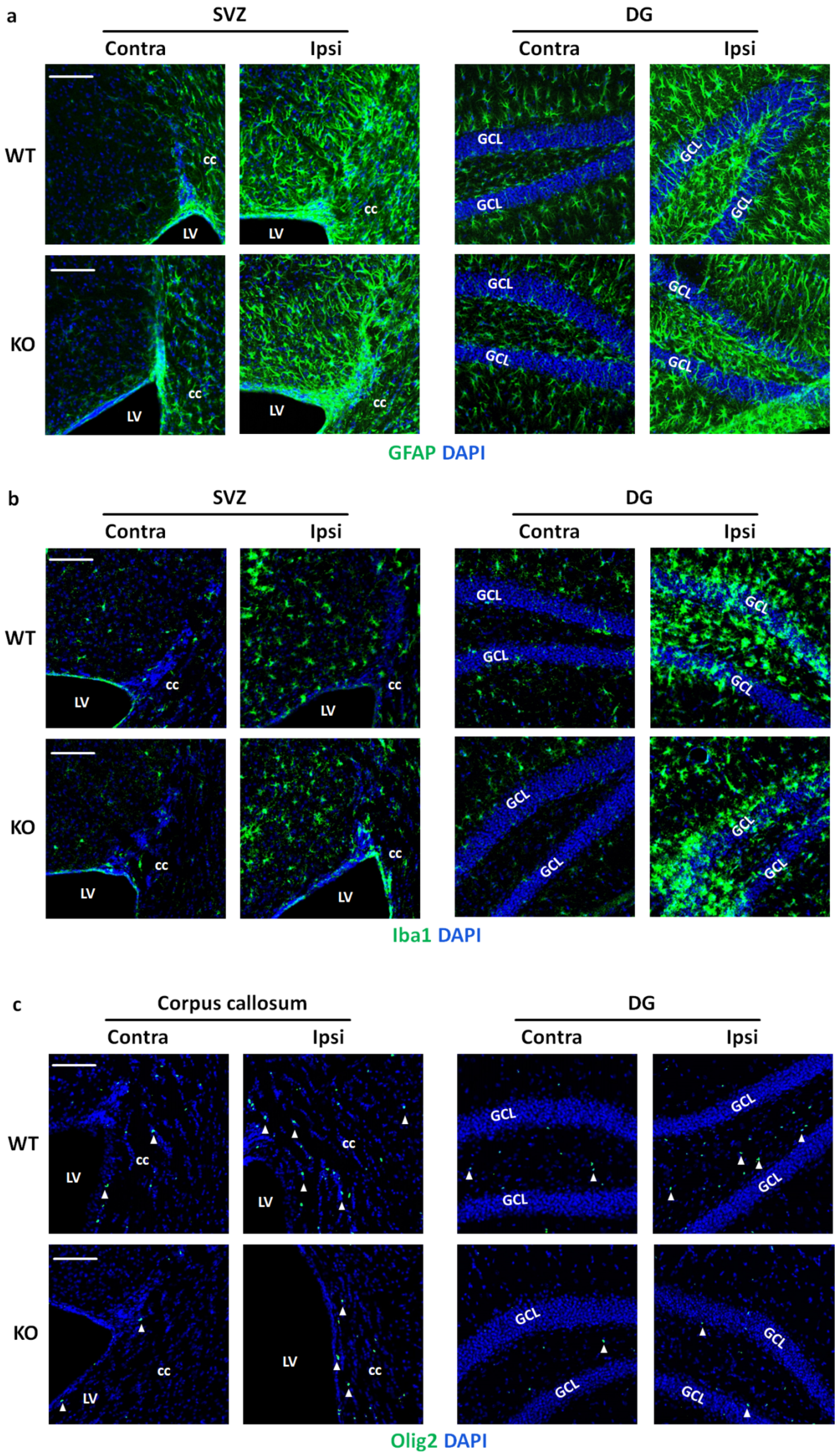

Supplementary Figure 3 (to be continued)

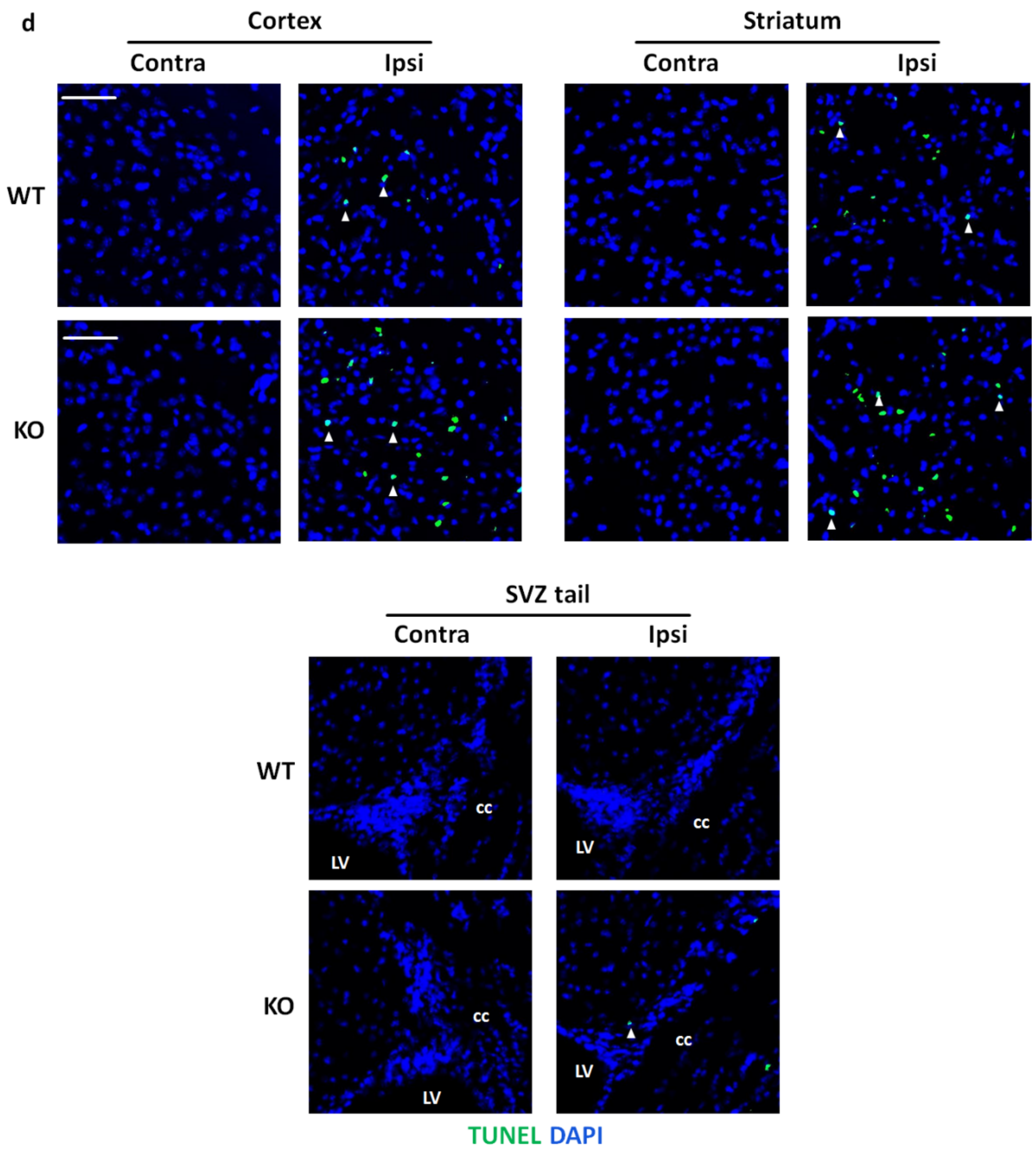

Supplementary Figure 3 (continued)

Supplementary Figure 3 HI injury caused glia cell stimulation and apoptosis in both RBM3 WT and KO mice.

a-c. Representative immunofluorescent staining of reactive astrocytes (GFAP+) **(a)**, microglia (Iba1+) **(b)** and oligodendrocyte precursors (Olig2+) **(c)** in the SVZ (corpus callosum for Olig2+) and DG as well as adjacent regions of RBM3 WT and KO animals treated with HI and recovered for 7 days. For each section, both contralateral and ipsilateral images were acquired under the same setting parameters of exposure period and gain value. Scale bar: 100 µm. LV: lateral ventricle; cc: corpus callosum; GCL: granular cell layer. **d.** Representative immunofluorescent TUNEL staining in the ischemic cores in the ipsilateral (Ipsi) side of cerebral cortex, striatum and SVZ tail of RBM3 WT and KO animals treated with HI and recovered for 7 days. The corresponding contralateral side (Contra) was represented as negative control. LV: lateral ventricle; cc: corpus callosum. Scale bar: 100 µm.

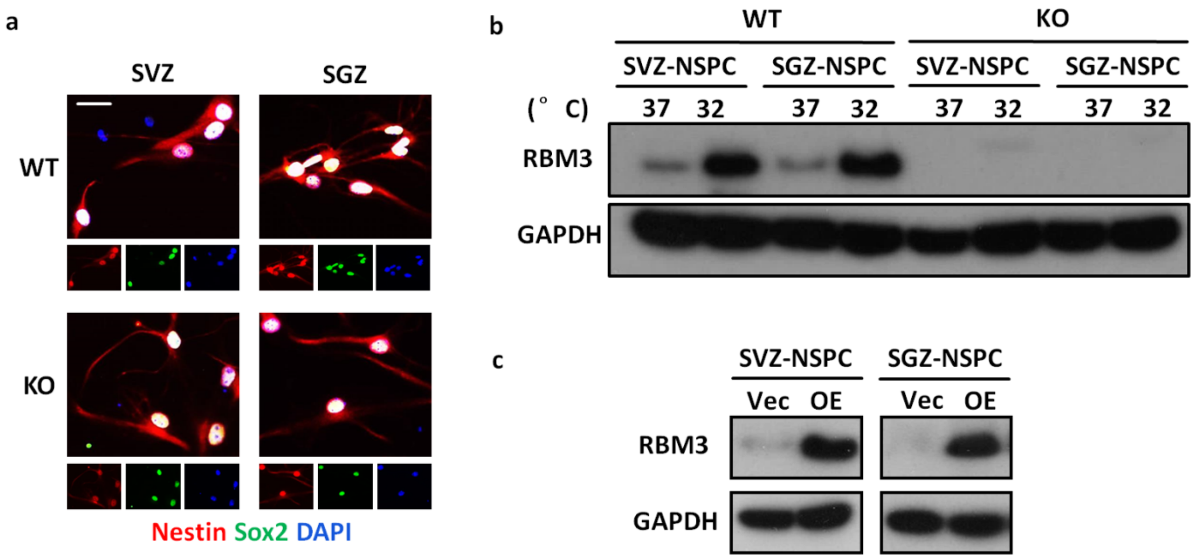

Supplementary Figure 4 RBM3 expression in cultured NSPCs.

a. Representative immunofluorescent staining of nestin and Sox2 in cultured RBM3 WT or KO NSPCs. Nestin (red), Sox2 (green) and DAPI (blue) were merged. Scale bar: 25 μm. **b.** Representative Western blot of RBM3 expression after hypothermic treatment in cultured WT NSPCs. **c.** Representative Western blot of RBM3 expression in WT NSPCs which were transfected with pCEP4 or pCEP4-RBM3 plasmids by electroporation. Vec: empty vector; OE: RBM3 overexpression.

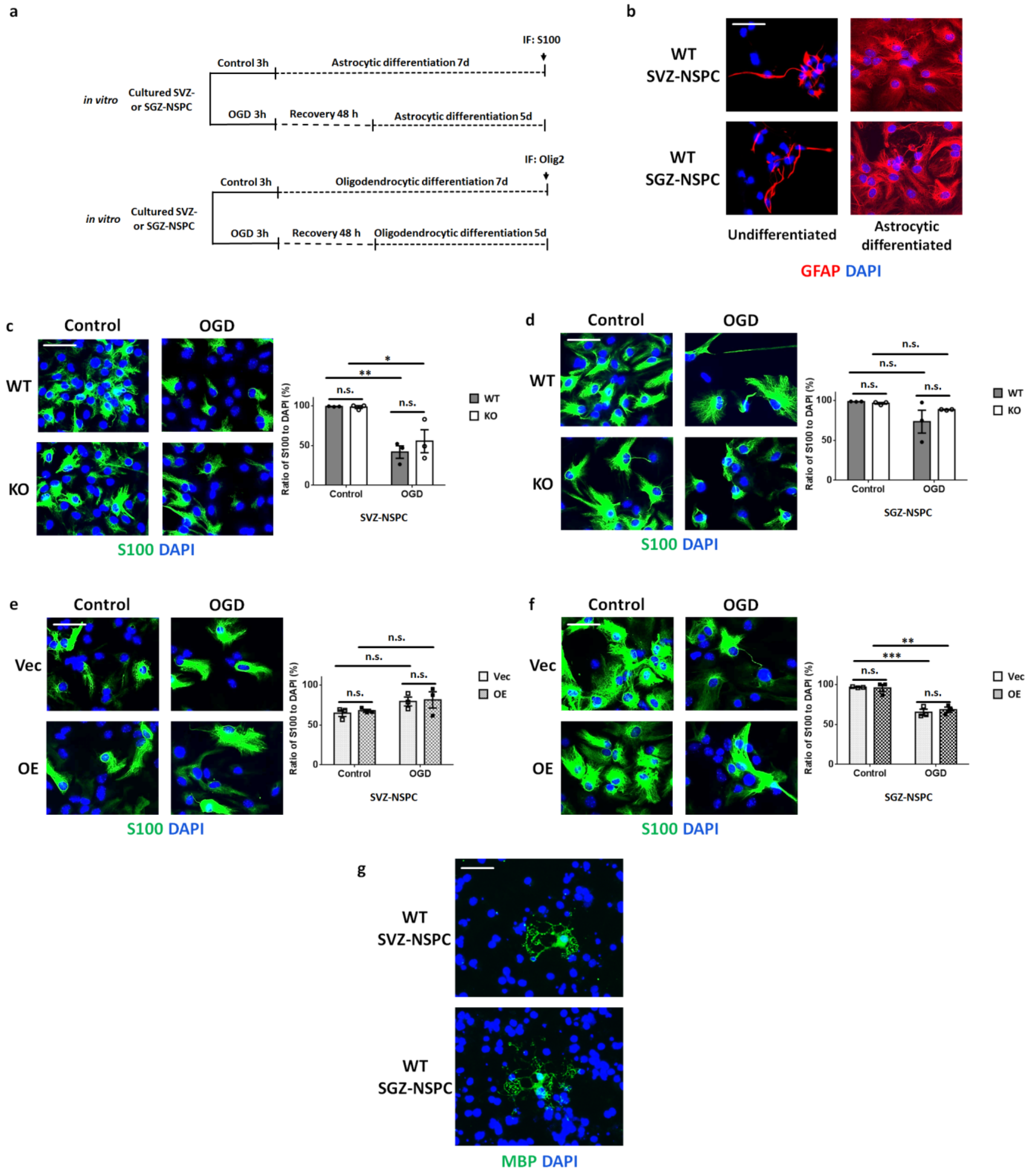

Supplementary Figure 5 (to be continued)

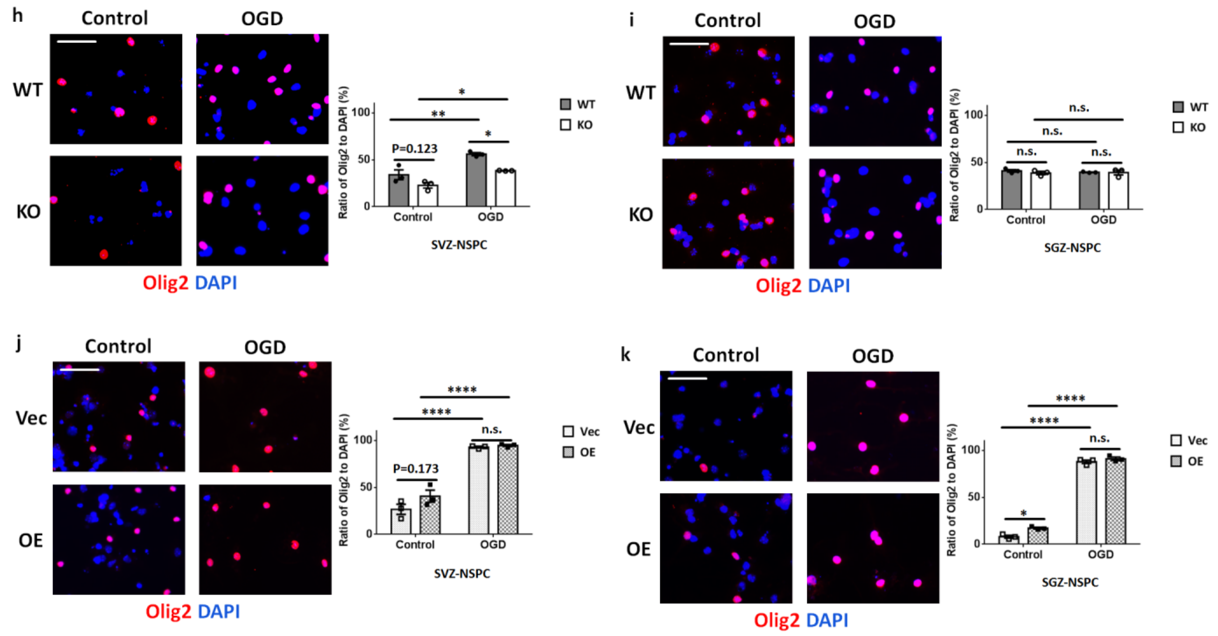

Supplementary Figure 5 (continued)

Supplementary Figure 5 RBM3 did not significantly affect glial differentiation of SVZ-NSPC and SGZ-NSPC *in vitro*.

a. Illustration of *in vitro* glial differentiation assay. NSPCs were cultured in astrocytic or oligodendrocytic differentiation medium for 7 days (control), or first challenged with OGD and then reoxygenated in NSPC complete culture medium for the first 2 days, followed by switching to astrocytic or oligodendrocytic differentiation medium for 5 days (OGD). OGD: oxygen-glucose deprivation; IF: immunofluorescence. **b.** Representative immunostaining of GFAP in undifferentiated or 7-day astrocytic differentiated WT NSPCs derived from SVZ or SGZ. Scale bar: 50 µm. **c-f.** RBM3 WT and KO NSPCs from SVZ **(c)** or SGZ **(d)**, and WT NSPCs transfected with empty vector (Vec) or RBM3 overexpressing vector (OE) with SVZ **(e)** or SGZ **(f)** origins were used in astrocytic differentiation assay. NSPSc were stained with glia cell marker S100 and the ratio of immunoreactive cells to DAPI positive cells were calculated (three independent experiments, n=3). Representative images were presented. Scale bar: 50 µm. Two-way ANOVA was used for statistical analysis. n.s. not significant; * p<0.05; ** p<0.01; *** p<0.001. **g.** Representative immunostaining of oligodendrocyte marker MBP in 7-day oligodendrocytic differentiated WT NSPCs derived from SVZ or SGZ. Scale bar: 50 µm. **h-k** RBM3 WT and KO NSPCs from SVZ **(h)** or SGZ **(i)**, and WT NSPCs transfected with empty vector (Vec) or RBM3 overexpressing vector (OE) with SVZ **(j)** or SGZ **(k)** origins were used in oligodendrocytic differentiation assay. NSPSc were stained with oligodendrocyte precursor cell marker Olig2 and the ratio of immunoreactive cells to DAPI positive cells were calculated (three independent experiments, n=3). Representative images were presented. Scale bar: 50 µm. Two-way ANOVA was used for statistical analysis. n.s. not significant; * p<0.05; ** p<0.01; **** p<0.0001. All data are presented in SEM.

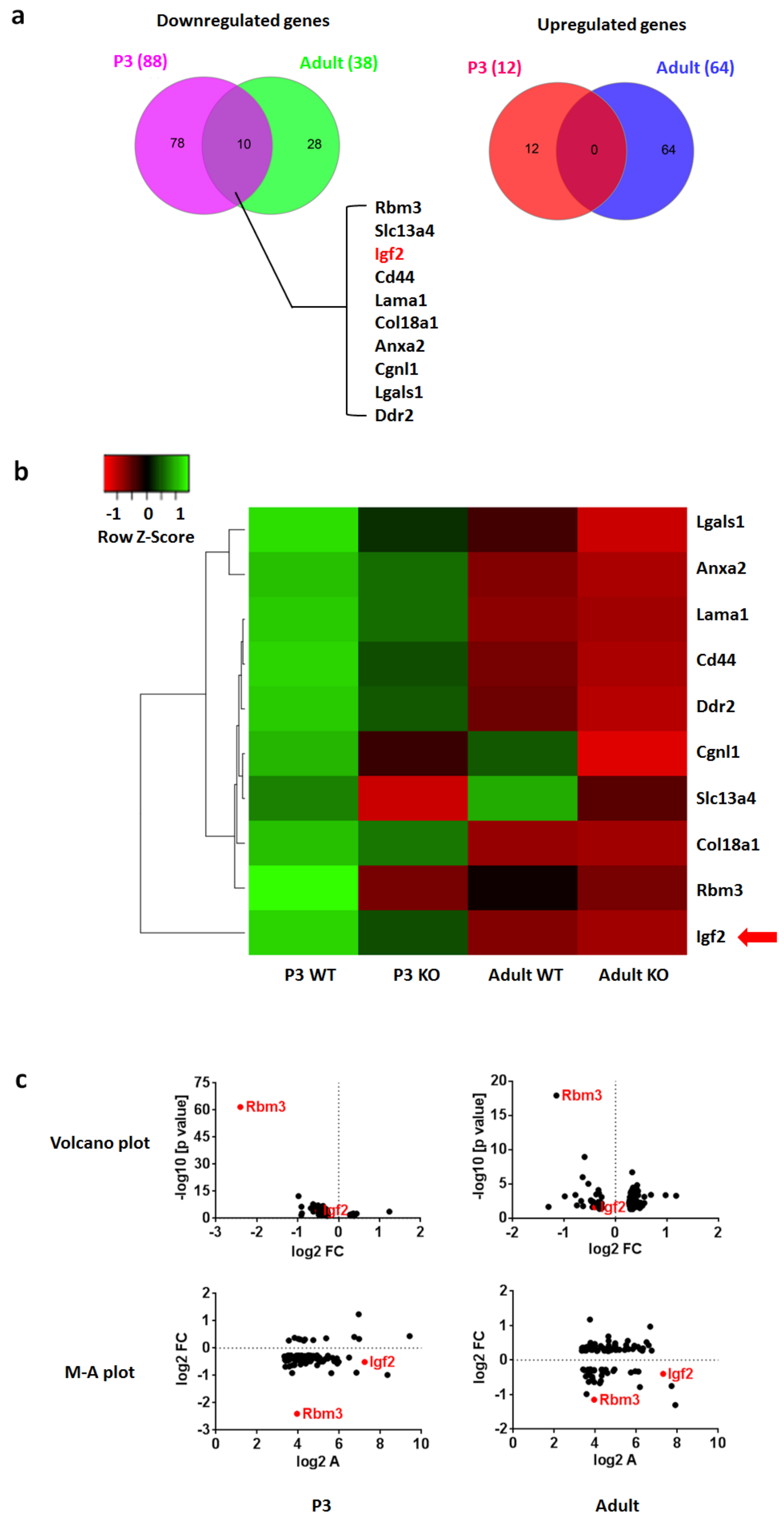

Supplementary Figure 6 (to be continued)

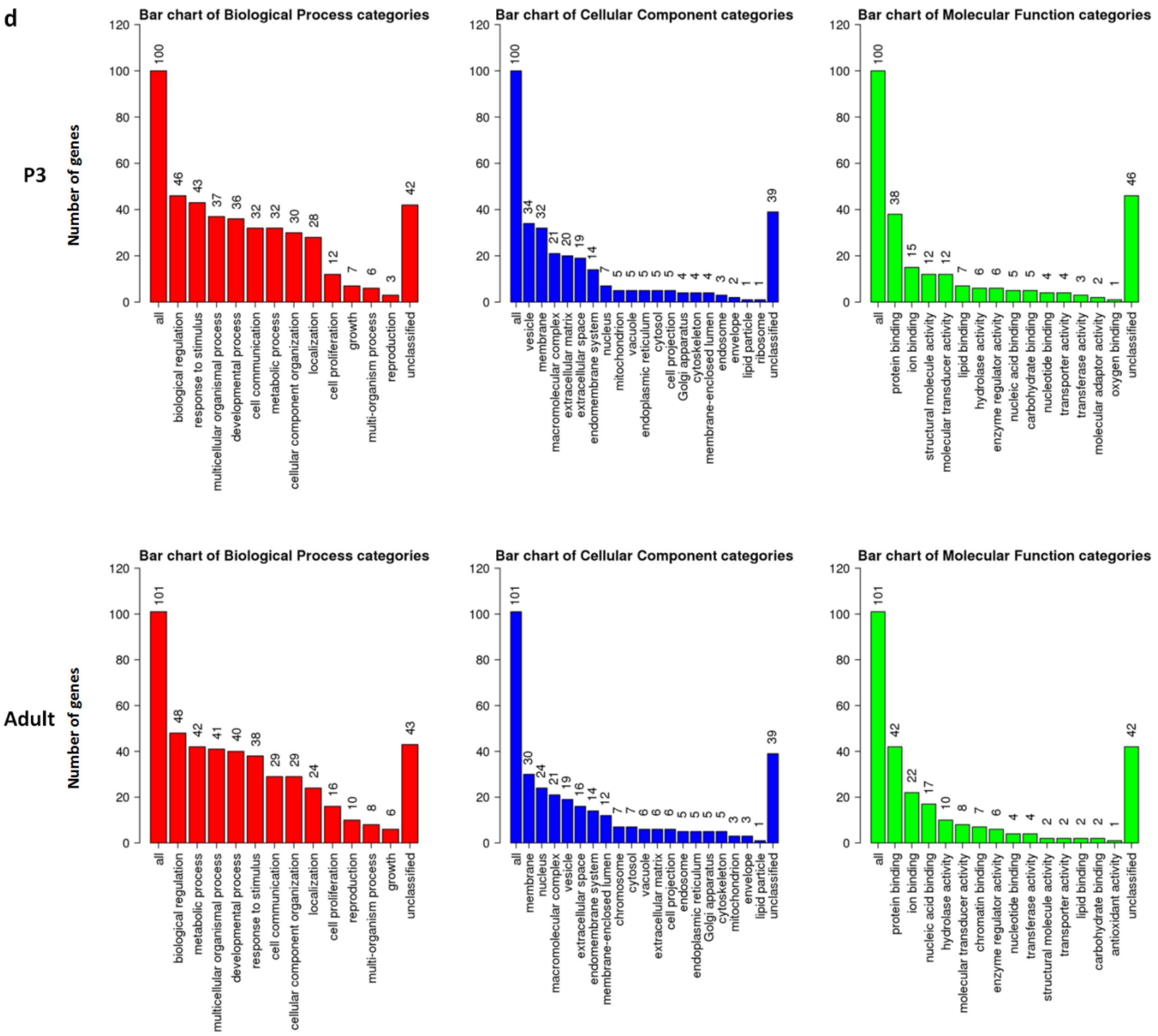

Supplementary Figure 6 (continued)

Supplementary Figure 6 RNA-seq analysis of RBM3 WT or KO hippocampi from postnatal day 3 (P3) or adult mice.

Differentially expressed genes (DEGs) were filtered with the following cutoffs: log2A > 3.32 (Average counts per million > 10); |log2 FC| > 0.26 (|Fold change| > 1.2) and unadjusted P value < 0.05.

a. Venn Diagrams of commonly downregulated or upregulated DEGs from P3 (KO vs WT) and adult (KO vs WT) lists. Ten commonly downregulated genes are listed, and *Igf2* is highlighted. **b.** Heatmap of the ten common DEGs generated by their Reads Per Kilobase Million (RPKM). Clustering distance was measured by Euclidean method. **c.** Volcano plot and M-A plot of all DEGs in P3 and adult samples, respectively. *Rbm3* and *Igf2* were highlighted. FC: fold change; A: average counts per million (average CPM). **d.** Gene set enrichment analysis (GSEA) in the three categories of biological process, cellular components and molecular functions of all DEGs in P3 and adult samples, respectively.

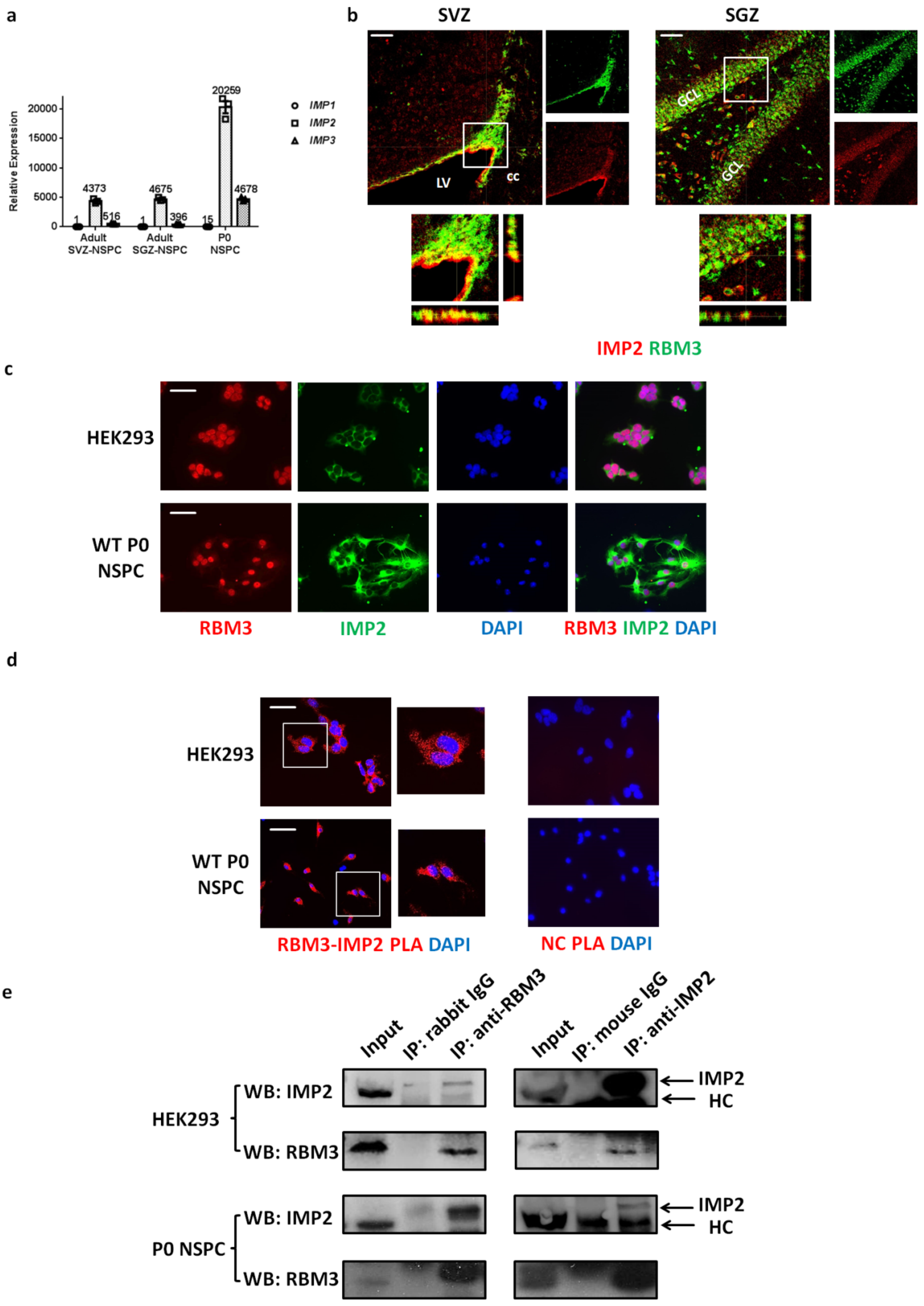

Supplementary Figure 7 (to be continued)

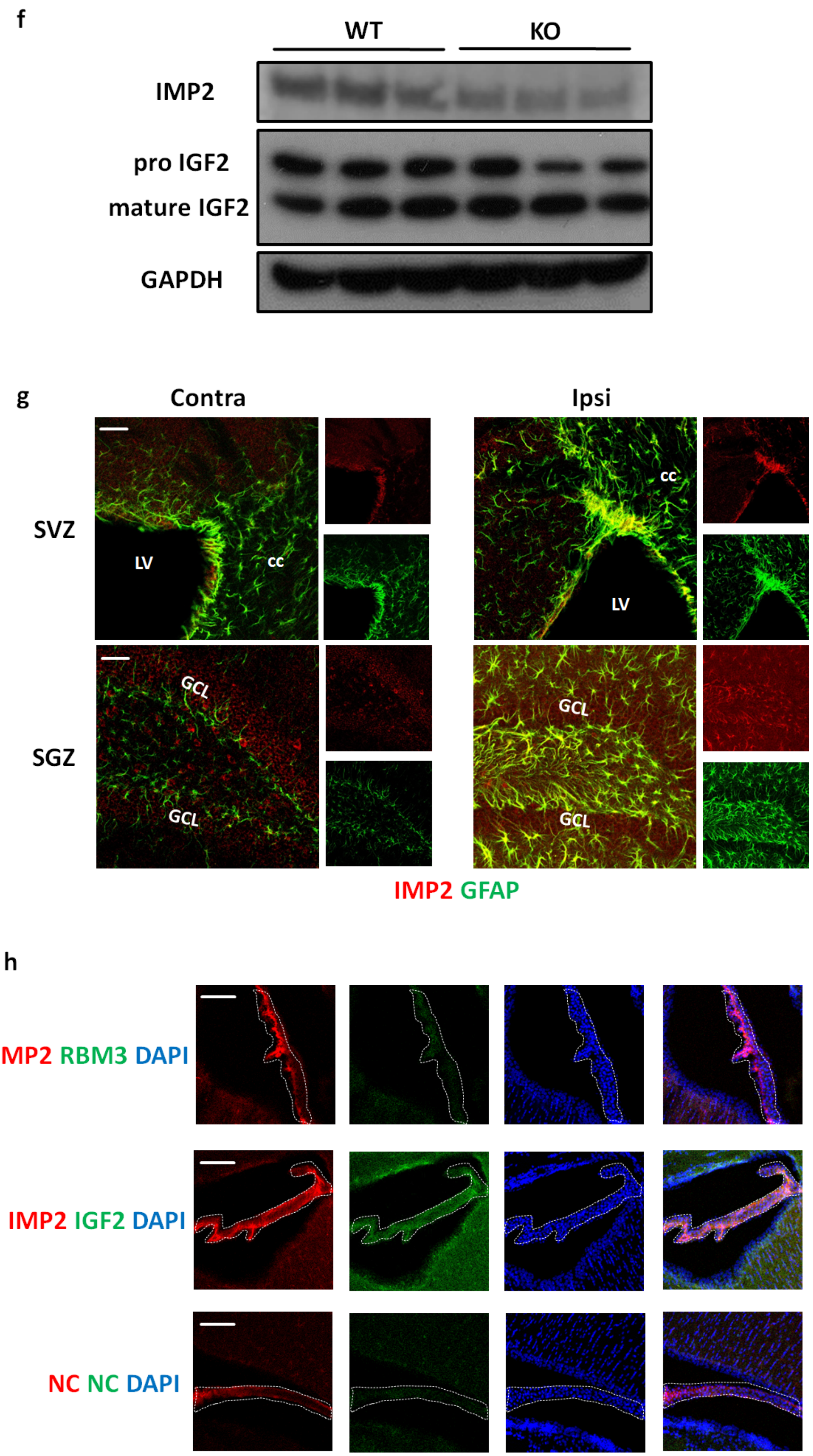

Supplementary Figure 7 (continued)

Supplementary Figure 7 Additional evidences of the involvement of IMP2-IGF2 pathway in RBM3 function on NSPC proliferation.

a. Quantitative RT-PCR of *IMP1*, *IMP2* and *IMP3* mRNA expressions in cultured adult SVZ-NSPC and SGZ-NSPCs. NSPCs from the whole brain of postnatal day 0 (P0) mouse was used as a positive control (three independent experiments, n=3). *IMP1*, *IMP2* and *IMP3* mRNA level was normalized to *GAPDH* by $2^{-\Delta\Delta CT}$ method. Relative amount of mRNA was listed on top of each column. All data are presented in SEM. **b.** Representative immunofluorescent staining of RBM3 and IMP2 in the SVZ and DG of adult WT mouse brain. Orthogonal view confirmed the co-localization of RBM3 and IMP2. Scale bar: 50 µm. LV: lateral ventricle; cc: corpus callosum; GCL: granular cell layer. **c.** Immunofluorescent co-staining of RBM3 and IMP2 in HEK 293 and WT P0 NSPC. Scale bar: 25 µm. **d.** Proximity ligation assay of endogenous RBM3 and IMP2 in HEK 293 and WT P0 NSPC. NC: negative control omitting both primary antibodies. Scale bar: 25 µm. **e.** CoIP of endogenous RBM3 and IMP2 in HEK293 and P0 NSPC. Rabbit polyclonal RBM3 antibody and mouse monoclonal IMP2 antibody were used for immunoprecipitation, respectively. Rabbit IgG was used as negative control for RBM3 antibody, mouse IgG was used as negative control for IMP2 antibody. HC: heavy chain of IgG used for immunoprecipitation. **f.** Western blot of IMP2 and IGF2 expression in whole brain lysate from adult WT or KO mice without treatment (three animals per group, n=3). Dual bands were observed for IGF2 at around 20kDa and 10kDa, indicating pro IGF2 and mature IGF2, respectively. **g.** Representative immunofluorescent staining of IMP2 and GFAP in the SVZ and DG of adult WT mice after HI injury plus 7 days recovery. LV: lateral ventricle; cc: corpus callosum; GCL: granular cell layer. Contra: contralateral (uninjured side); ipsi: ipsilateral (injured side). Scale bar: 50 µm. **h.** RBM3, IMP2 and IGF2 expressions in the choroid plexus of adult WT mice. NC: Negative control with secondary antibodies only but without primary antibody. Scale bar: 100 µm.

Figure 5e

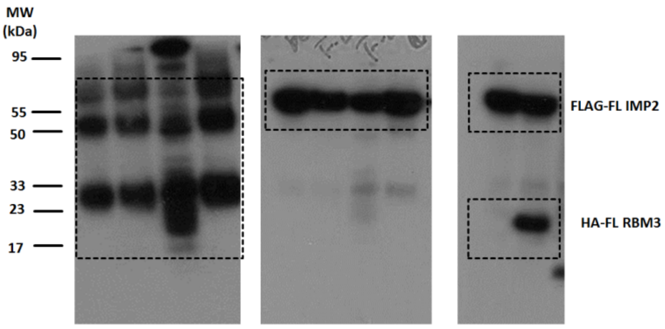

Figure 5f

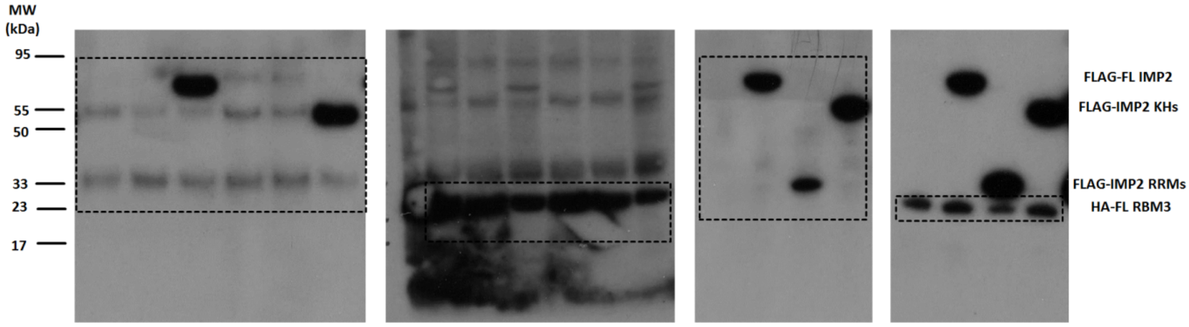

Figure 5g

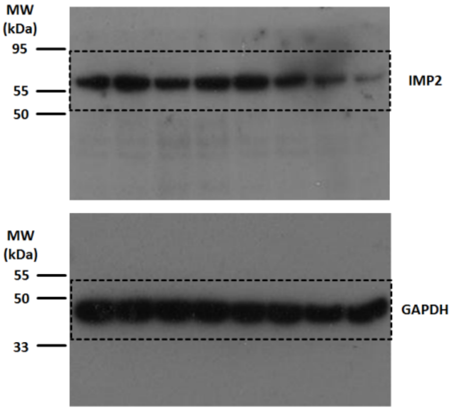

Figure 6e

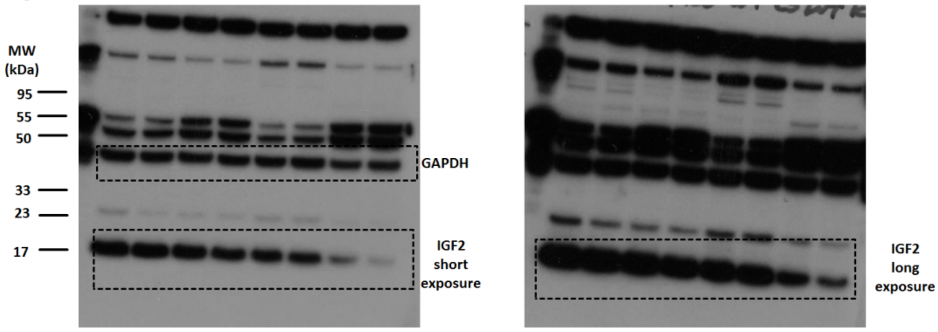

Supplementary Figure 8 (to be continued)

Supplementary Figure 4b

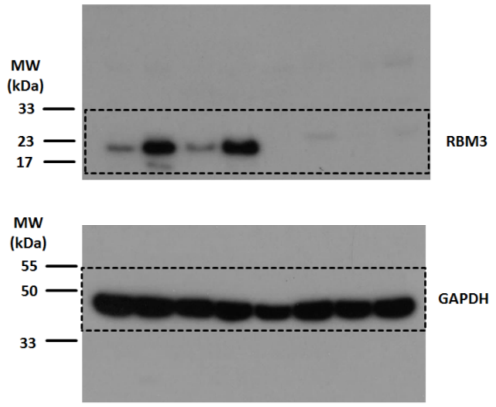

Supplementary Figure 4c

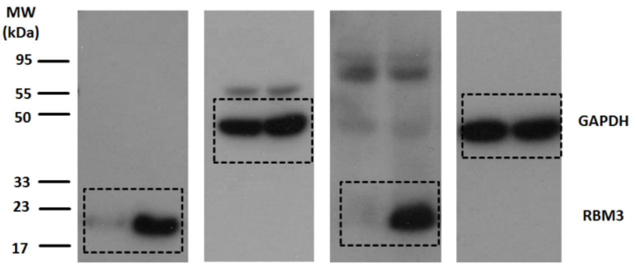

Supplementary Figure 7e

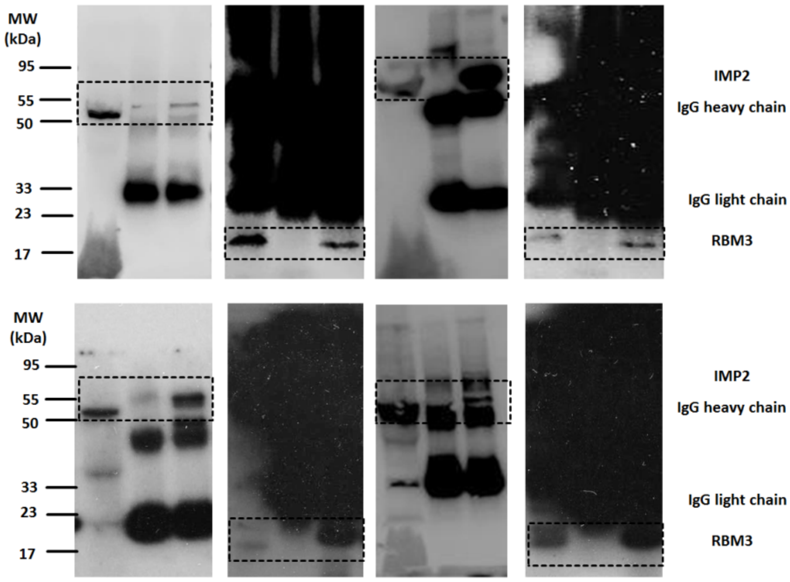

Supplementary Figure 7f

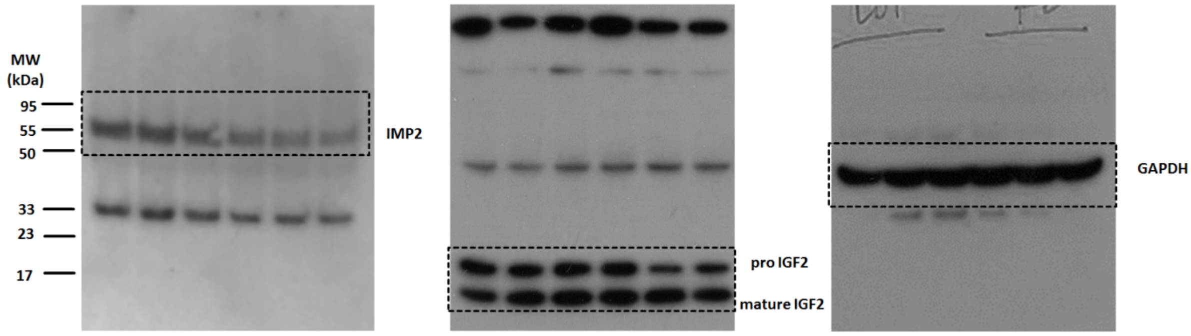

Supplementary Figure 8 (continued)

Supplementary Figure 8 Uncropped Western blot images.
