# Supplementary material for: RBM3 promotes neurogenesis in a niche-dependent manner via IMP2-IGF2 signaling pathway after hypoxic-ischemic brain injury
Source: Nat Commun. 2019 Sep 4;10:3983. doi: 10.1038/s41467-019-11870-x (PMC6726629; doi:10.1038/s41467-019-11870-x)
Supplement: Supplementary file 3 — Reporting Summary [file 41467_2019_11870_MOESM3_ESM.pdf]

nature research

Corresponding author(s): Xinzhou Zhu and Sven Wellmann

Last updated by author(s): Jul 22, 2019

Reporting Summary

Nature Research wishes to improve the reproducibility of the work that we publish. This form provides structure for consistency and transparency in reporting. For further information on Nature Research policies, see Authors & Referees and the Editorial Policy Checklist .

Statistics

For all statistical analyses, confirm that the following items are present in the figure legend, table legend, main text, or Methods section.

n/a	Confirmed	
☐	☒	The exact sample size (*n*) for each experimental group/condition, given as a discrete number and unit of measurement
☐	☒	A statement on whether measurements were taken from distinct samples or whether the same sample was measured repeatedly
☐	☒	The statistical test(s) used AND whether they are one- or two-sided<br>*Only common tests should be described solely by name; describe more complex techniques in the Methods section.*
☒	☐	A description of all covariates tested
☐	☒	A description of any assumptions or corrections, such as tests of normality and adjustment for multiple comparisons
☒	☐	A full description of the statistical parameters including central tendency (e.g. means) or other basic estimates (e.g. regression coefficient) AND variation (e.g. standard deviation) or associated estimates of uncertainty (e.g. confidence intervals)
☐	☒	For null hypothesis testing, the test statistic (e.g. *F*, *t*, *r*) with confidence intervals, effect sizes, degrees of freedom and *P* value noted<br>*Give P values as exact values whenever suitable.*
☒	☐	For Bayesian analysis, information on the choice of priors and Markov chain Monte Carlo settings
☒	☐	For hierarchical and complex designs, identification of the appropriate level for tests and full reporting of outcomes
☒	☐	Estimates of effect sizes (e.g. Cohen's *d*, Pearson's *r*), indicating how they were calculated

Our web collection on statistics for biologists contains articles on many of the points above.

Software and code

Policy information about availability of computer code

Data collection	*Provide a description of all commercial, open source and custom code used to collect the data in this study, specifying the version used OR state that no software was used.*
Data analysis	*Provide a description of all commercial, open source and custom code used to analyse the data in this study, specifying the version used OR state that no software was used.*

For manuscripts utilizing custom algorithms or software that are central to the research but not yet described in published literature, software must be made available to editors/reviewers. We strongly encourage code deposition in a community repository (e.g. GitHub). See the Nature Research guidelines for submitting code & software for further information.

Data

Policy information about availability of data

All manuscripts must include a data availability statement. This statement should provide the following information, where applicable:

- Accession codes, unique identifiers, or web links for publicly available datasets
- A list of figures that have associated raw data
- A description of any restrictions on data availability

The raw data of RNA-seq are available on NCBI BioProject (Project ID: PRJNA529585, [https://www.ncbi.nlm.nih.gov/bioproject/?term=PRJNA529585]).). The source data underlying Figures 1b, 1c, 1e-1h, 2b-2e, 3b-3i, 4b, 4c, 4e-4h, 5b, 6a-6d, 6f, 6g, 7a-7c and Supplementary Figures 1a, 1b, 1e-1h, 2a-2f, 5c-5f, 5h-5k, 7a are provided as a Source Data file. Uncropped blots are shown in Supplementary Figure 8. All the other raw data are available from the authors upon request. A reporting summary for this article is available as a Supplementary Information file.

Field-specific reporting

Please select the one below that is the best fit for your research. If you are not sure, read the appropriate sections before making your selection.

- [x] Life sciences
- [ ] Behavioural & social sciences
- [ ] Ecological, evolutionary & environmental sciences

For a reference copy of the document with all sections, see nature.com/documents/nr-reporting-summary-flat.pdf

Life sciences study design

All studies must disclose on these points even when the disclosure is negative.

Sample size
For animal experiments, five animals for sham or HI+28d and ten animals for HI+7d group were used per group to meet the minimal requirements of statistical analysis. For in vitro experiments, at least triplicates were used to meet the minimal requirements of statistical analysis.

Data exclusions
The mortality rate of adult mice during hypoxia exposure in HI model was around 8%. The animals which died during hypoxia exposure were excluded from further experiments. All animals survived from hypoxia exposure were used for further histological and biochemical analysis. In HI+7d group, all ten animals per group were subjected to histological analysis. Six out of these ten animals were randomly chosen for immunostaining and subsequent cell quantification, in order to reduce the workload of quantification, and simultaneously fulfill the requirement for statistical analysis. In sham and HI+28d groups, no animal was excluded from analysis.

Replication
All replications were successful.

Randomization
RBM3 WT or KO mice were randomly allocated into sham, HI+7d or HI+28d groups.

Blinding
Microscopy and stereological cell quantifications were performed in a blinded way, as this method is sensitive to experimenter's biases. Sample preparation, histological and biochemical experiments were not performed in a blinded way. qPCR and WB values were normalized with internal control, thus blinded experiment is not necessary.

Reporting for specific materials, systems and methods

We require information from authors about some types of materials, experimental systems and methods used in many studies. Here, indicate whether each material, system or method listed is relevant to your study. If you are not sure if a list item applies to your research, read the appropriate section before selecting a response.

Materials & experimental systems

n/a	Involved in the study	
☐	☒	Antibodies
☐	☒	Eukaryotic cell lines
☒	☐	Palaeontology
☐	☒	Animals and other organisms
☒	☐	Human research participants
☒	☐	Clinical data

Methods

n/a	Involved in the study	
☒	☐	ChIP-seq
☒	☐	Flow cytometry
☒	☐	MRI-based neuroimaging

Antibodies

Antibodies used

WB: Western blot, IP: immunoprecipitation, IF: immunofluorescent staining, IP: immunoprecipitation
mouse normal IgG (Santa Cruz, sc-2025), 1:50 for IP
anti-BrdU (Abcam, ab6326), rat monoclonal, 1:250 for IF
anti-Dcx (Millipore, AB2253), guinea pig polyclonal, 1:1000 for IF
anti-FLAG (DDK) (OriGene, TA100011), mouse monoclonal, 1:1000 for WB, 1:200 for IP
anti-GAPDH (Abcam, ab8245), mouse monoclonal, 1:2000 for WB
anti-GFAP (DAKO, Z0334), rabbit polyclonal, 1:1000 for IF
anti-HA (OriGene, TA100012), mouse monoclonal, 1:1000 for WB, 1:200 for IP
anti-Iba1 (Wako, 019-19741), rabbit polyclonal, 1:200 for IF
anti-IGF2 (Abcam, ab9574), rabbit polyclonal, 1:1000 for WB, 1:250 for IF
anti-IMP2 (IGF2BP2) (Abnova, H00010644-M01), mouse monoclonal, 1:500 for WB, 1:125 for IP, 1:100 for IF
anti-MAP2 (Cell Signaling Technology, #4542), rabbit polyclonal, 1:100 for IF
anti-MBP (Boehringer Mannheim, 1118099), mouse monoclonal, 1:100 for IF
anti-nestin (Novus, NBP1-02419), rabbit polyclonal, 1:200 for IF
anti-NeuN (Millipore, MAB377), mouse monoclonal, 1:250 for IF
anti-Olig2 (Millipore, AB9610), rabbit polyclonal, 1:200 for IF

	anti-RBM3 (Proteintech, 14363-1-AP), rabbit polyclonal, 1:1000 for WB, 1:37.5 for IP, 1:100 for IF<br>anti-S100 (Abcam, ab7852), mouse monoclonal, 1:50 for IF<br>anti-Sox2 (R&D, MAB2018), mouse monoclonal, 1:250 for IF
Validation	All antibodies were validated in mouse samples on manufacturers' websites, with cited references if applicable. Anti-RBM3 antibody was also validated in our brain samples from RBM3 WT and KO mice (Supplementary Figure 4b).

Eukaryotic cell lines

Policy information about cell lines

Cell line source(s)	Human embryonic kidney 293 cell line (HEK293) was purchased from Merck Sigma-Aldrich (Catalogue number: 85120602-1VL).
Authentication	HEK293 cell line used in this study was not authenticated.
Mycoplasma contamination	HEK293 cell line used in this study was tested negatively for mycoplasma contamination.
Commonly misidentified lines<br>(See ICLAC register)	None.

Animals and other organisms

Policy information about studies involving animals; ARRIVE guidelines recommended for reporting animal research

Laboratory animals	Mouse (Mus musculus); C57BL/6 background, RBM3 gene wildtype (WT) or knockout (KO); only male animals were used in this study; postnatal day 0 (P0) and 2-3 months old
Wild animals	The study did not involve wild animals.
Field-collected samples	The study did not involve samples collected from the field.
Ethics oversight	All animal experiments were approved by the veterinary office of Basel city (German name: Kantonales Veterinäramt BS; authorization number 2064 and 2652).

Note that full information on the approval of the study protocol must also be provided in the manuscript.
