# Supplementary material for: RBM3 promotes neurogenesis in a niche-dependent manner via IMP2-IGF2 signaling pathway after hypoxic-ischemic brain injury
Source: Nat Commun. 2019 Sep 4;10:3983. doi: 10.1038/s41467-019-11870-x (PMC6726629; doi:10.1038/s41467-019-11870-x)
Supplement: Supplementary file 4 — Description of Additional Supplementary Files [file 41467_2019_11870_MOESM4_ESM.docx]

**Description of Additional Supplementary Files**

File Name: Supplementary Data 1
Description: Summary of RNA quality and total reads number

File Name: Supplementary Data 2
Description: Differentially expressed genes (DEGs) in RBM3 KO hippocampi from postnatal day 3 (P3) mice compared to WT, ranked by fold change. The cutoff settings were described in the table, and a full list without cutoffs was also provided.

File Name: Supplementary Data 3
Description: Differentially expressed genes (DEGs) in RBM3 KO hippocampi from adult mice compared to WT, ranked by fold change. The cutoff settings were described in the table, and a full list without cutoffs was also provided.

File Name: Supplementary Data 4
Description: Information for primary antibodies and primers
