# Supplementary material for: RBM3 promotes neurogenesis in a niche-dependent manner via IMP2-IGF2 signaling pathway after hypoxic-ischemic brain injury
Source: Nat Commun. 2019 Sep 4;10:3983. doi: 10.1038/s41467-019-11870-x (PMC6726629; doi:10.1038/s41467-019-11870-x)
Supplement: Supplementary file 2 — Peer Review [file 41467_2019_11870_MOESM2_ESM.pdf]

Reviewers' comments:

Reviewer #1 (Remarks to the Author):

This is an interesting article. The authors used RBM3 mutant mice and NSPCs isolated from AVZ and SGZ of these mice to show that RBM3 promotes ischemia induced neurogenesis and it does so by interacting with IMP2 and stimulating IGF2 release. Although interesting and potentially impactful, I found a number of significant issues.

First, although the conclusion that RBM3 is important for HI-induced neurogenesis is generally supported by the data, the proposed mechanism is not sufficiently supported.
(1) The physical interaction between RBM3 and IMP2 was only assessed by co-transfection of overexpression plasmids. This is a key data therefore should be validated for endogenous proteins in NSPCs. It will be especially interesting to see whether this interaction is present or stronger in SGZ NSPCs vs SVZ NSPCs
(2) The RNA-seq was done in WT and KO hippocampi without HI and IGF2 came out as one of the top hits. However, in the context of HI, IGF2 may not be a top hit.
(3) The IGF2 release is concluded without strong data support. IGF amount was measured in supernatant in cultured NSPCs where they saw difference and in CSF where they did not see difference. The IGF2 protein levels should be analyzed in in vivo in NSPCs and neurons using co-staining instead of just showing only IGF2 staining (Figure 6H). Similarly IMP2 staining should be done too. These data will show whether the elevated IGF2 in response to HI is really intrinsic to NSPCs or in the niche or both

Second, all in vivo cell quantifications were performed in thin 25 um sections and data are presented as cell number/mm2 (mm square). This does not meet the gold standard for adult neurogenesis analysis. Because adult NSPCs and new neurons are in small numbers and also because the shape of adult hippocampus changes from dorsal to ventral, it is important to use unbiased stereology with appropriate guard zones for in vivo quantification. The data should be presented both in total cell number/DG and the number of cells/mm3 (mm cubic). The volume of the DG should be measured using optical fractionator as a parameter too. I consider this insufficiency in methodology is a significant issue. As reviewed in a recent article on human adult hippocampal neurogenesis (Kempermann Cell stem cell 2018), appropriate quantitative method can lead to different conclusions about adult neurogenesis.

Third, the manuscript depends on colocalization of cell markers for data analysis, however most images are in poor quality. Better quality images used. To demonstrate colocalization or a lack of, single channel confocal images and orthogonal confocal images need to be included

Fourth, I am also concerned with several seemingly standard methodology used here. Because many labs have used these methods, what these methods can resolve and major issues they encounter are well known too.
(1) The neuronal, astrocyte, and oligodendrocyte differentiation data appear good but how they did this is questionable. (Figure S4). It is rare to see such high % oligodendrocyte differentiation from wild type adult NSPCs (nearly 50%). The neuronal differentiation was done by withdrawal growth factor, but such treatment leads to cell death in adult NSPCs even though it can be used in embryonic NSPCs. More details are needed for evaluation.

(2) In Figure 3B and 3C, they quantify cell fate after RBM3 overexpression but they did not distinguish transfected versus non-transfected cells. In fact in this study, they used electroporation to introduce plasmid into NSPCs. It is well known that the efficiency is not very high (20%-60%) and cell death of transfected cells is significant. I am concerned what they quantified.

(3) Fourth, the There is no description in RNA-seq data quality, reads, and bioinformatics.

Specific points:
(1) Figure S1B: why are WT sections darker in color compared to KO?
(2) What is the point of showing mild injury in Figure S2A? No additional data or discussion seems to relate to this.
(3) MAP2+ neurons in vitro are not necessarily "mature" neurons.
(4) Figure S6C showing colocalization should be in main figure. It is a key data
(5) Authors need to eliminate "trended to" language. If there is no statistical significance then they should state so.
(6) "data not shown" should not be included since there is always room for supplemental data.

Reviewer #2 (Remarks to the Author):

In this study mechanisms underlying potential therapeutic effects of hypothermia for neonatal hypoxia-ischemia (HI) were studied in mice. Specifically, a mouse model in which RNA-binding motif protein 3 (RBM3), a neuroprotective protein induced by cold stress was ablated. The authors indicate that RMB3 promotes cell genesis in the dentate gyrus (DG) but not the subventricular zone (SVZ) after HI. The underlying mechanism is reported to be an interaction between RBM3 and IGF2 binding protein, selectively, in the subgranular zone (SGZ) progenitor cells. The following issues should be addressed:

1. The basic premise of the paper is that ablation of RBM3 affects adult dentate gyrus neurogenesis, but not subventricular zone following HI. Specifically, cell proliferation and neurogenesis, apoptosis and IGF2 levels under pathological conditions such as HI are only increased in the WT mice but not the KO mice. However, under basal conditions there is no difference between wildtype (WT) and knockout (KO) mice. To support these claims the authors provide several pieces of data that are not altogether convincing.
2. Brain weight and Nissl stained sections are provided of WT and KO as evidence that there are no structural differences. The sample sections shown in Suppl Figure 1, are not very neat or clear and the anterior-posterior levels do not match precisely. Systematic volumetric analysis of the at least the structures of interest in the present study (hippocampus, subventricular zone) would help support this claim.
3. The images shown in Figure 1 of the SVZ and DG are oversaturated and become rapidly pixelated when zoomed in on. There are no low power over overviews of the areas of interest or high magnification images of double-labeled cells (e.g. BrdU-Sox2).
4. Total BrdU cell counts are not provided for any of the conditions, only double-labeling of Sox2 and BrdU. These data are based on 4 sections per mouse, with 5 mice per condition and is reported as a density measure but the Methods lack details as to how these data were obtained. The distance between the sections is 150 microns indicating that the quantified area (~600 micron) represents only a fraction of the entire SVZ or SGZ. It might be premature to base such a strong conclusion, that only SGZ but not SVZ is affected by RBM3 ablation in HI, on such a restricted sampling of tissue sections. It would be better to perform a detailed and comprehensive analysis of BrdU labeled cells throughout the anterior-posterior extent of the structure as well as volume measurements to determine how the HI affected the structures.
5. In Supplementary Figure 2a (line 90 main text) it is stated that the infarct volume was significantly greater in KO than in WT brains. It is not clear how the infarction volume was calculated. In some sections almost half the brain is missing (severe HI) whereas for mild the left -right difference is not detectable and it is not clear how the authors know which side of the brain is right or left, ipsi- or contralateral to the injury, there is no landmark or notch detectable in the tissue samples shown.
6. The immunofluorescence images in Figures 1 and 2 are oversaturated. It is difficult to see where the cells are located. The double-labeling analysis is not very convincing. The NeuN – BrdU staining

(Figure 2D,E) does not show clearly delineated cells and nuclei. Similar concerns apply to Figure 4C (apoptosis) and Figure 6G (IGF2 intensity levels).

7. The statistical tests used are mentioned in the manuscript but there is no report of the F scores for the ANOVAs or the degrees of freedom in the tests, making it difficult to assess the validity of the data reported in the study.

Reviewers' comments:

Reviewer #1 (Remarks to the Author):

This is an interesting article. The authors used RBM3 mutant mice and NSPCs isolated from AVZ and SGZ of these mice to show that RBM3 promotes ischemia induced neurogenesis and it does so by interacting with IMP2 and stimulating IGF2 release. Although interesting and potentially impactful, I found a number of significant issues.

RESPONSE: We highly appreciate the reviewer's encouraging and helpful comments for improving our work. Please find our responses to each comment below.

First, although the conclusion that RBM3 is important for HI-induced neurogenesis is generally supported by the data, the proposed mechanism is not sufficiently supported. (1) The physical interaction between RBM3 and IMP2 was only assessed by co-transfection of overexpression plasmids. This is a key data therefore should be validated for endogenous proteins in NSPCs. It will be especially interesting to see whether this interaction is present or stronger in SGZ NSPCs vs SVZ NSPCs

RESPONSE: We agree with the reviewer that the physical interaction between endogenous RBM3 and IMP2 needs to be demonstrated in NSPCs. We now provide novel CoIP data for neonatal NSPCs (P0) in addition to the HEK293 cell data (Supplementary Figure S6E). Our novel data confirm a direct interaction of RBM3 and IMP2 in neonatal NSPCs (Supplementary Figure S6E).
In adult NSPCs, CoIP experiments are not feasible due to the low expression levels of both RBM3 and IMP2. This is because their expressions decrease strongly from embryonic stage to adulthood as described in the manuscript [1-3]. Thus, in these cells we used proximity ligation assay (PLA, Figure 5B) which shows not only co-localization but also protein-protein interactions [4]. Hence, the advantage of PLA is to overcome the limitation of CoIP in case of low protein expression. Interestingly, in SGZ-NSPCs there are more RBM3-IMP2 interactions than in SVZ-NSPCs after OGD (Figure 5B). Furthermore, we could demonstrate RBM3-IMP2 interaction using PLA in adult SVZ and SGZ *in vivo* (Figure 5C).

(2) The RNA-seq was done in WT and KO hippocampi without HI and IGF2 came out as one of the top hits. However, in the context of HI, IGF2 may not be a top hit.

RESPONSE: The reviewer is right that the initial finding of a reduction of IGF2 expression in RBM3 KO mice came from RNA-seq data of normal hippocampus. The demonstration of the involvement of IGF2 in the context of HI was shown in further experiments presented in this manuscript confirming the critical role of IGF2 expression in this context. There was no need to repeat the RNA-seq experiments in the context of HI. Our finding that RBM3 interacts with IMP2 for stimulating IGF2 release after HI in SGZ-NSPCs but not SVZ-NSPCs is based on these experiments, not on RNA-seq data.

(3) The IGF2 release is concluded without strong data support. IGF amount was measured in supernatant in cultured NSPCs where they saw difference and in CSF where they did not see difference. The IGF2 protein levels should be analyzed in in vivo in NSPCs and neurons using co-staining instead of just showing only IGF2 staining (Figure 6H). Similarly IMP2 staining should be done too. These data will show whether the elevated IGF2 in response to HI is really intrinsic to NSPCs or in the niche or both

RESPONSE: We think that increased presence of IGF2 in the supernatant of cultured NSPCs is actually "strong data support" for IGF2 release by NSPCs. Whereas the observed lack of difference in CSF may have the following reason. The choroid plexus produces CSF including IGF2 [5, 6]. However, RBM3 is not expressed in choroid plexus in adult WT mice (Supplementary Figure S6H). Thus, RBM3 is unlikely to regulate IGF2 level in CSF, which may explain why SVZ-NSPC proliferation was not affected in RBM3 KO mice (Supplementary Figure S6H). Actually, the IGF2 response in SVZ-NSPC is niche-dependent but not intrinsic to themselves, as they reacts with IGF2 produced in CSF but not IGF2 secreted by itself [5, 7].
We have taken up the suggestion of the reviewer to perform co-staining of IGF2 and Sox2 in NSPCs *in vivo*. These novel data confirm increased expression of IGF2 in the SGZ neurogenic niche after HI in WT, but not in RBM3 depleted mice (Figure 6I), and no difference in the SVZ niche (Figure 6H). They also show that NSPC is not the only cell type expressing IGF2 in these niches consistent with the known strong activation of glial cells after HI. The IGF2 response in SGZ-NSPC is intrinsic to themselves, but not niche-dependent [5, 7], thus not affected by adjacent glia cell activation.

Second, all in vivo cell quantifications were performed in thin 25 um sections and data are presented as cell number/mm2 (mm square). This does not meet the gold standard for adult neurogenesis analysis. Because adult NSPCs and new neurons are in small numbers and also because the shape of adult hippocampus changes from dorsal to ventral, it is important to use unbiased stereology with appropriate guard zones for in vivo quantification. The data should be presented both in total cell number/DG and the number of cells/mm3 (mm cubic). The volume of the DG should be measured using optical fractionator as a parameter too. I consider this insufficiency in methodology is a significant issue. As reviewed in a recent article on human adult hippocampal neurogenesis (Kempermann Cell stem cell 2018), appropriate quantitative method can lead to different conclusions about adult neurogenesis.

RESPONSE: We agree with the reviewer that unbiased stereology is superior for *in vivo* cell quantifications. We have now repeated our analysis using this method, the details of sample preparation and cell quantification methods are given in the revised method section of the manuscript. The new data are presented in Fig 1B and 1C, Fig 2B-E, Supplementary Figure S1E, S1F, S2C and S2D and essentially confirm our earlier findings.

Third, the manuscript depends on colocalization of cell markers for data analysis, however most images are in poor quality. Better quality images used. To demonstrate colocalization or a lack of, single channel confocal images and orthogonal confocal images need to be included

RESPONSE: We now have improved image quality, showing single channels and orthogonal images to show co-localization. Double positive cells were imaged using z-stack function. Stacked images were acquired every 1 µm throughout the section and are presented in orthogonal view to confirm co-localization. For examples see Fig 1B and 1C, Fig 2B-E.

Fourth, I am also concerned with several seemingly standard methodology used here. Because many labs have used these methods, what these methods can resolve and major issues they encounter are well known too.
(1) The neuronal, astrocyte, and oligodendrocyte differentiation data appear good but how they did this is questionable. (Figure S4). It is rare to see such high % oligodendrocyte differentiation from wild type adult NSPCs (nearly 50%). The neuronal differentiation was

done by withdrawal growth factor, but such treatment leads to cell death in adult NSPCs even though it can be used in embryonic NSPCs. More details are needed for evaluation.

RESPONSE: We think the discrepancy noticed by the reviewer is due to the definition of oligodendrocyte differentiation. We focused on Olig2+ oligodendrocyte progenitor cells (OPCs) in our work. We observed a comparable high differentiation ratio as other researchers did, as Olig2+ OPCs can be easily induced from NSCs (Figure 1C and D in reference [8]). Instead, it is difficult to induce mature MBP+ oligodendrocyte (Supplementary Figure S4G). And with just few MBP+ cells it is impossible to evaluate RBM3 effects on mature oligodendrocyte differentiation.
Growth factor withdrawal is a standard method for neuronal differentiation from NSC, and it is widely used by others in both embryonic and adult NSPC culture [9, 10]. We agree that adult NSPCs are more vulnerable than embryonic NSPCs, but cell death caused by mitogen withdrawal is unfortunately inevitable in current *in vitro* differentiation system [9].

(2) In Figure 3B and 3C, they quantify cell fate after RBM3 overexpression but they did not distinguish transfected versus non-transfected cells. In fact in this study, they used electroporation to introduce plasmid into NSPCs. It is well known that the efficiency is not very high (20%-60%) and cell death of transfected cells is significant. I am concerned what they quantified.

RESPONSE: For these experiments, we had optimized the transfection procedure and routinely obtained transfection rates around 90% before subjecting cells to ODG or differentiation experiments (see Figure 2 in RESPONSE shown below). Thus, we have not further distinguished between transfected and non-transfected cells. It is highly unlikely that the low number of non-transfected cells would affect the outcome of these experiments.

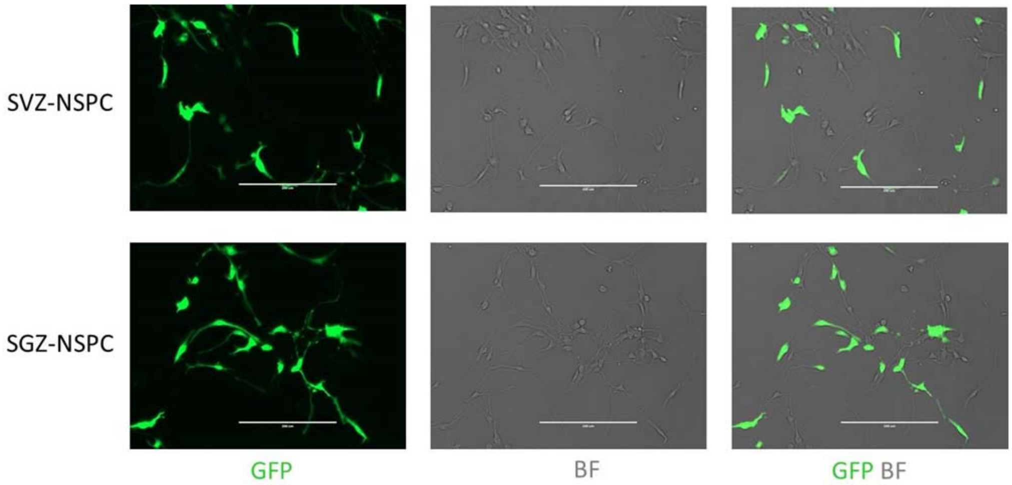

Figure 1 in RESPONSE: Wildtype SVZ-NSPCs and SGZ-NSPCs were transfected with pmaxGFP control plasmid (Lonza), incubated in 12-well plate overnight, spun down and then seeded in coated chamber slide for another 24 h incubation. GFP fluorescence was visualized in live cells. BF: bright field. Scale bar: 200 µm. Transfection efficiency was approximately 60%.

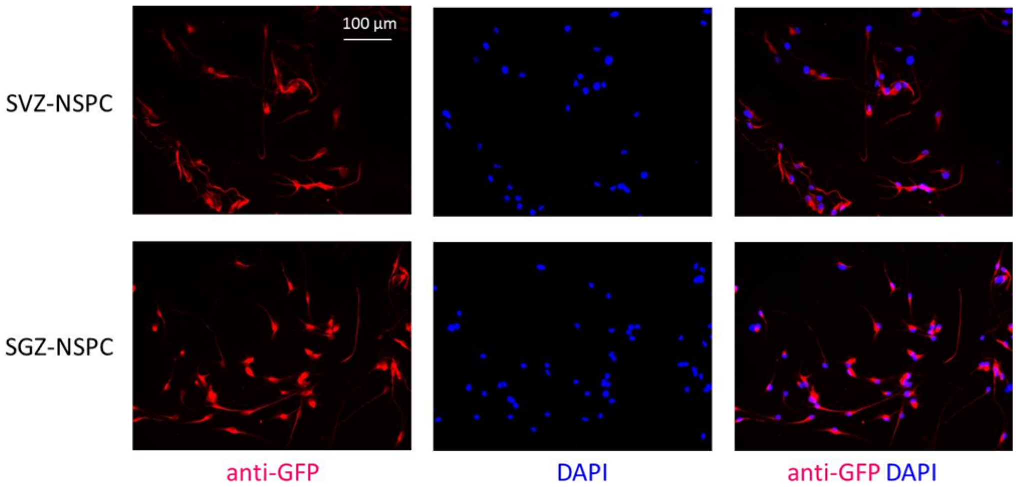

Figure 2 in RESPONSE: Cells from Figure 1 in RESPONSE were cultured for additional 24 h, totally 72 h after transfection. Then cells were fixed by 4% PFA and incubated with anti-GFP primary antibody. Alexa 568 secondary antibody was used to detect GFP. DAPI was counterstained. Scale bar: 100 µm. Around 90% cells were GFP positive.

The details of the optimized protocol are given below and in the manuscript:
The commercial Lonza Nucleofector protocol with Mouse Neural Stem Cell Nucleofector Kit is an optimized electroporation method for NSPC transfection with higher efficiency (>60%). Other researchers used the same method to transfect mouse adult NSPCs [11, 12]. Briefly, $5x10^6$ NSPCs were transfected with 10 µg plasmid DNA with Lonza system. After transfection, cells were resuspended in 500 µL complete culture medium and transferred to 12-well plate overnight for recovery. As NSPCs do not attach to uncoated plate and overnight incubation is too short to form neurospheres, most of the cells remained as single cells. To get rid of dead cells, they were spun down with low speed centrifugation and seeded at a density of $1x10^4$ viable cells/mL into poly-L-lysine coated chamber slide for another 24h incubation. At this stage (48h after transfection) we observed 60% positive cells based on the transfection of a GFP-expressing plasmid (Figure 1 in RESPONSE). After changing the medium and additional 24h of incubation (72 h after transfection), around 90% cells were GFP positive (Figure 2 in RESPONSE). This is due to the continuing expression of target gene and the washout of dead cells.

(3) Fourth, the There is no description in RNA-seq data quality, reads, and bioinformatics.

RESPONSE: We have now included RNA integrity number (RIN) and total reads data in Supplementary Table S1 in the revised manuscript. As we observed very few differentially expressed genes (DEGs) using adjusted p value <0.05, we did not set a p value cutoff in our first draft. Now we revised the cutoff conditions with unadjusted p value < 0.05 and |fold change| > 1.2 to have a more stringent threshold and subsequently a shortened DEG list. We added additional bioinfomatic analysis in Supplementary Figure S5A-S5D including Venn's diagram, heatmap of overlapped DEGs between P3 and adult mice, volcano and M-A plots, and gene set enrichment analysis (GSEA). Our main target gene *Igf2* was still found on the top list.

Specific points:
(1) Figure S1B: why are WT sections darker in color compared to KO?

RESPONSE: This was due to a technical problem with the previous staining. We have repeated cresyl violet staining and replaced the images.

(2) What is the point of showing mild injury in Figure S2A? No additional data or discussion seems to relate to this.

RESPONSE: We have revised Supplementary Figure S2A and now we show the representative images of average injury, and also used a new method for quantification of infarction volume according to references [13, 14]. Despite identical manipulation in the HI model, there is always a variation of injury from animal to animal.

(3) MAP2+ neurons in vitro are not necessarily “mature” neurons.

RESPONSE: We now designate MAP2 as neuronal marker instead of mature neuronal marker.

(4) Figure S6C showing colocalization should be in main figure. It is a key data

RESPONSE: As suggested by the reviewer we have moved Supplementary Figure S6C to the main manuscript (new Figure 5C).

(5) Authors need to eliminate “trended to” language. If there is no statistical significance then they should state so.

RESPONSE: We replaced “tended to” with “remained unchanged” as suggested by the reviewer.

(6) “data not shown” should not be included since there is always room for supplemental data.

RESPONSE: As suggested by the reviewer, we deleted “data not shown” and have added novel images showing GFAP+ NSPCs (Supplementary Figure S4B) and rare TUNEL+ cells in the lateral tail of SVZ (Supplementary Figure S2H). Consequently, two new references demonstrating the presence of GFAP in adult NSPCs are now added to the manuscript [15, 16] (in the manuscript refenrence [20, 21]). That is the reason why we did not use GFAP as astrocytic marker in differentiation assay.

Reviewer #2 (Remarks to the Author):

In this study mechanisms underlying potential therapeutic effects of hypothermia for neonatal hypoxia-ischemia (HI) were studied in mice. Specifically, a mouse model in which RNA-binding motif protein 3 (RBM3), a neuroprotective protein induced by cold stress was ablated. The authors indicate that RMB3 promotes cell genesis in the dentate gyrus (DG) but not the subventricular zone (SVZ) after HI. The underlying mechanism is reported to be an interaction between RBM3 and IGF2 binding protein, selectively, in the subgranular zone (SGZ) progenitor cells. The following issues should be addressed:

RESPONSE: We sincerely thank you for your helpful comments and suggestions. Our responses to each comment are listed below.

1. The basic premise of the paper is that ablation of RBM3 affects adult dentate gyrus neurogenesis, but not subventricular zone following HI. Specifically, cell proliferation and neurogenesis, apoptosis and IGF2 levels under pathological conditions such as HI are only increased in the WT mice but not the KO mice. However, under basal conditions there is no difference between wildtype (WT) and knockout (KO) mice. To support these claims the authors provide several pieces of data that are not altogether convincing.

RESPONSE: No specific comment.

2. Brain weight and Nissl stained sections are provided of WT and KO as evidence that there are no structural differences. The sample sections shown in Suppl Figure 1, are not very neat or clear and the anterior-posterior levels do not match precisely. Systematic volumetric analysis of the at least the structures of interest in the present study (hippocampus, subventricular zone) would help support this claim.

RESPONSE: We agree with the reviewer that systematic volumetric analysis of the SVZ and DG is desirable. We have now estimated SVZ and DG volumes according to published methods [17, 18] (technical details can be found in the Methods of the revised manuscript) and the new data are presented in Supplementary Figure S1B.

3. The images shown in Figure 1 of the SVZ and DG are oversaturated and become rapidly pixelated when zoomed in on. There are no low power over overviews of the areas of interest or high magnification images of double-labeled cells (e.g. BrdU-Sox2).

RESPONSE: The images in Figure 1 and 2 have been replaced with images of improved quality. Orthogonal confocal images are now included for the confirmation of double-labeled cells.

4. Total BrdU cell counts are not provided for any of the conditions, only double-labeling of Sox2 and BrdU. These data are based on 4 sections per mouse, with 5 mice per condition and is reported as a density measure but the Methods lack details as to how these data were obtained. The distance between the sections is 150 microns indicating that the quantified area (~600 micron) represents only a fraction of the entire SVZ or SGZ. It might be premature to base such a strong conclusion, that only SGZ but not SVZ is affected by RBM3 ablation in HI, on such a restricted sampling of tissue sections. It would be better to perform a detailed and comprehensive analysis of BrdU labeled cells throughout the anterior-posterior extent

of the structure as well as volume measurements to determine how the HI affected the structures.

RESPONSE: We followed your suggestions and re-analyzed our samples accordingly. A detailed description of the new method is given in the method section of the revised manuscript. In brief, we sectioned 25 µm thick, serial coronal sections and picked every twelfth section (300 µm interval). We stained and quantified all positive cells in four sections for SVZ (approximately between +1.2 mm and 0.0 mm bregma) and eight sections for SGZ (approximately between -1.2 mm and -3.6 mm bregma) per animal. The volumes of SVZ and DG (mm3) were estimated on adjacent sections with published methods [17, 18]. Both total cell number and cell density (cell number/mm3) are now presented. Total BrdU counting is now included as suggested (Supplementary Figure S2C and S2D). The new data confirm our previous conclusions.

5. In Supplementary Figure 2a (line 90 main text) it is stated that the infarct volume was significantly greater in KO than in WT brains. It is not clear how the infarction volume was calculated. In some sections almost half the brain is missing (severe HI) whereas for mild the left -right difference is not detectable and it is not clear how the authors know which side of the brain is right or left, ipsi- or contralateral to the injury, there is no landmark or notch detectable in the tissue samples shown.

RESPONSE: We agree with the reviewer that the quantification of the infarction volume was suboptimal in the original manuscript. We now use a new quantification method similar to the one used in references [13, 14]. The details are given in the Methods part of the revised manuscript. We confirmed our conclusion that in KO brains the infarction volume was greater, but there was no change of SVZ or DG volumes (Supplementary Figure S2A and S2B). Nevertheless, it is clear that despite identical application of the HI injury model there is a substantial individual variability in the outcome from animal to animal.
As for the labeling of the left and right hemispheres, the ischemic hemisphere could always be unambiguously identified by glial activation (in immunofluorescent staining) and neuronal loss (in both immunofluorescent and cresyl violet staining) which were clearly present even in mild injury.

6. The immunofluorescence images in Figures 1 and 2 are oversaturated. It is difficult to see where the cells are located. The double-labeling analysis is not very convincing. The NeuN – BrdU staining (Figure 2D,E) does not show clearly delineated cells and nuclei. Similar concerns apply to Figure 4C (apoptosis) and Figure 6G (IGF2 intensity levels).

RESPONSE: We have replaced Figure 1 and 2 with better confocal images showing the orthogonal view for double-labeled cells. We have replaced images of TUNEL staining in Figure 4C with better quality images, and also the images of IGF2 staining and co-staining with Sox2 in Figure 6H and 6I.

7. The statistical tests used are mentioned in the manuscript but there is no report of the F scores for the ANOVAs or the degrees of freedom in the tests, making it difficult to assess the validity of the data reported in the study.

RESPONSE: We have now included F-score and degree of freedom (DF) in our statistics in Supplementary Table S4.

References:

1. Pilotte, J., et al., *Developmentally regulated expression of the cold-inducible RNA-binding motif protein 3 in euthermic rat brain.* Brain Res, 2009. **1258**: p. 12-24.
2. Fujii, Y., Y. Kishi, and Y. Gotoh, *IMP2 regulates differentiation potentials of mouse neocortical neural precursor cells.* Genes Cells, 2013. **18**(2): p. 79-89.
3. Nishino, J., et al., *A network of heterochronic genes including Imp1 regulates temporal changes in stem cell properties.* Elife, 2013. **2**: p. e00924.
4. Soderberg, O., et al., *Direct observation of individual endogenous protein complexes in situ by proximity ligation.* Nat Methods, 2006. **3**(12): p. 995-1000.
5. Ferron, S.R., et al., *Differential genomic imprinting regulates paracrine and autocrine roles of IGF2 in mouse adult neurogenesis.* Nat Commun, 2015. **6**: p. 8265.
6. Terauchi, A., et al., *Retrograde fibroblast growth factor 22 (FGF22) signaling regulates insulin-like growth factor 2 (IGF2) expression for activity-dependent synapse stabilization in the mammalian brain.* Elife, 2016. **5**.
7. Bracko, O., et al., *Gene expression profiling of neural stem cells and their neuronal progeny reveals IGF2 as a regulator of adult hippocampal neurogenesis.* J Neurosci, 2012. **32**(10): p. 3376-87.
8. Havrda, M.C., et al., *Id2 mediates oligodendrocyte precursor cell maturation arrest and is tumorigenic in a PDGF-rich microenvironment.* Cancer Res, 2014. **74**(6): p. 1822-32.
9. Walker, T.L. and G. Kempermann, *One mouse, two cultures: isolation and culture of adult neural stem cells from the two neurogenic zones of individual mice.* J Vis Exp, 2014(84): p. e51225.
10. Johe, K.K., et al., *Single factors direct the differentiation of stem cells from the fetal and adult central nervous system.* Genes Dev, 1996. **10**(24): p. 3129-40.
11. Krampert, M., et al., *Smad7 regulates the adult neural stem/progenitor cell pool in a transforming growth factor beta- and bone morphogenetic protein-independent manner.* Mol Cell Biol, 2010. **30**(14): p. 3685-94.
12. Keravala, A., et al., *Long-term transgene expression in mouse neural progenitor cells modified with phiC31 integrase.* J Neurosci Methods, 2008. **173**(2): p. 299-305.
13. Llovera, G., et al., *Modeling stroke in mice: permanent coagulation of the distal middle cerebral artery.* J Vis Exp, 2014(89): p. e51729.
14. Swanson, R.A., et al., *A semiautomated method for measuring brain infarct volume.* J Cereb Blood Flow Metab, 1990. **10**(2): p. 290-3.
15. Imura, T., H.I. Kornblum, and M.V. Sofroniew, *The predominant neural stem cell isolated from postnatal and adult forebrain but not early embryonic forebrain expresses GFAP.* J Neurosci, 2003. **23**(7): p. 2824-32.
16. Garcia, A.D., et al., *GFAP-expressing progenitors are the principal source of constitutive neurogenesis in adult mouse forebrain.* Nat Neurosci, 2004. **7**(11): p. 1233-41.
17. Qiu, J., et al., *Regenerative response in ischemic brain restricted by p21cip1/waf1.* J Exp Med, 2004. **199**(7): p. 937-45.
18. Gonzalez, F.F., et al., *Erythropoietin enhances long-term neuroprotection and neurogenesis in neonatal stroke.* Dev Neurosci, 2007. **29**(4-5): p. 321-30.

Reviewers' comments:

Reviewer #1 (Remarks to the Author):

The revised manuscript has improved especially in terms of image quality.
My concern remains for in vivo cell quantifications. These analyses were still performed in thin 25 um sections without the use of guard zone and unbiased sampling, the key components for stereology. In fact the methods described for stereology is not really stereology.
Some more minor points:
Fig 6H and 6I will benefit from zoom in high magnification photos of positive cells
The authors rebutted my point about electroporation efficiency. The figure 1 in the response does not show 90% efficiency as claimed, but rather more like 60-70%. Therefore my points for original figure 3B and 3C about they did not distinguish transfected vs non-transfected cells remain.

Reviewer #2 (Remarks to the Author):

A more detailed stereological and and volumetric analysis using StereoInvestigator or Neurolucida would certainly be preferred. However, Image J if properly applied could provide adequate information. Upon re-examination of the methods and data I have some doubts as to whether that was done correctly, and about the statistical analyses. Attached please find my comments to consider.

Reviewer #1 (Remarks to the Author):

The revised manuscript has improved especially in terms of image quality.
My concern remains for in vivo cell quantifications. These analyses were still performed in thin 25 um sections without the use of guard zone and unbiased sampling, the key components for stereology. In fact the methods described for stereology is not really stereology.

RESPONSE: As suggested by the reviewer, we now have used the optical fractionator in Stereo Investigator software for cell quantification. Please see Methods part for detailed description.

Some more minor points:
Fig 6H and 6I will benefit from zoom in high magnification photos of positive cells

RESPONSE: We have added high magnification images in Figure 6h and 6i.

The authors rebutted my point about electroporation efficiency. The figure 1 in the response does not show 90% efficiency as claimed, but rather more like 60-70%. Therefore my points for original figure 3B and 3C about they did not distinguish transfected vs non-transfected cells remain.

RESPONSE: As the reviewer correctly mentioned, we observed 60-70% efficiency 24 h after transfection (as shown in Figure 1 in our previous RESPONSE). By allowing further overexpression for additional 24 h and washing out loosely attached cells with low viability, we observed 90% overexpressing cells (Figure 2 in our previous RESPONSE). Starting with these cells at this time point we did OGD or differentiation experiments.

Reviewer #2 (Remarks to the Author):

A more detailed stereological and volumetric analysis using StereoInvestigator or Neurolucida would certainly be preferred. However, Image J if properly applied could provide adequate information. Upon re-examination of the methods and data I have some doubts as to whether that was done correctly, and about the statistical analyses. Attached please find my comments to consider.

RESPONSE: We have now re-analyzed our samples using the optical fractionator in Stereo Investigator for cell quantification, and Cavalieri estimator probe for volume estimation. Please see Methods part for details.

Following are our response to specific points listed in the extra PDF file:

1. Volume measurements and BrdU counts.
We have switched to the Cavalieri estimator probe in Stereo Investigator software for volume estimation. The dentate gyrus volume includes granule cell layer (GCL) and hilus, but excludes the molecular layer. We have now added this information in Methods.

Although there is massive loss of neurons, the boundary of the ipsilateral DG granule cell layer (GCL) is still clear. When we measured the DG volume including hilus, there was no significant change between ipsilateral and contralateral.

We have added total BrdU counts of 28 days in the new Supplementary Figures S2e and S2f. Due to tissue damage, there is a bit folding where the blue arrow indicates, although it did not affect cell counting. We have replaced the representative image of BrdU+NeuN+ in the DG of WT ipsilateral site.

2. Statistical analysis. The sham data is the mean of sham contralateral and sham ipsilateral. As suggested, we have now used ‘repeated measures two-way ANOVA’ for the analysis of contralateral and ipsilateral groups from the same animal. We have also arranged the layout of source data in Supplementary Table 4, making it clearer that we included 5 sham animals and 6 HI animals per genotype (Figure 1b, 1c, 2b, 2c S2c and S2d).

REVIEWERS' COMMENTS:

Reviewer #2 (Remarks to the Author):

The authors have substantially improved the manuscript. Further clarification pertaining to the volume and density measurements used for the in vivo experiments would be helpful.
In their rebuttal the authors state in point 1: 'Although there is massive loss of neurons, the boundary of the ipsilateral DG granule cell layer (GCL) is still clear. When we measured the DG volume including hilus, there was no significant change between ipsilateral and contralateral.'
It is not quite clear what the structure consists of then. In the ipsilateral DG after the infarct, in Suppl figure 2d, HI + 7d the DAPI staining clearly shows there is a loss of cells in the granule cell layer. Is there a change in DG volume that should be considered when estimating the number of new neurons following infarct?

REVIEWERS' COMMENTS:

Reviewer #2 (Remarks to the Author):

The authors have substantially improved the manuscript. Further clarification pertaining to the volume and density measurements used for the in vivo experiments would be helpful.

In their rebuttal the authors state in point 1: ‘Although there is massive loss of neurons, the boundary of the ipsilateral DG granule cell layer (GCL) is still clear. When we measured the DG volume including hilus, there was no significant change between ipsilateral and contralateral.’

It is not quite clear what the structure consists of then. In the ipsilateral DG after the infarct, in Suppl figure 2d, HI + 7d the DAPI staining clearly shows there is a loss of cells in the granule cell layer. Is there a change in DG volume that should be considered when estimating the number of new neurons following infarct?

RESPONSE: As described in Methods, we measured the DG volume including granule cell layer (GCL) and hilus but excluding molecular layer (ML). NSPCs are located in subgranular zone (SGZ) in uninjured conditions, while after injury, quite a few proliferating and differentiating NSPCs are also present in GCL and hilus (Figure 1c, 2c and 2e). In contrast, in ML almost no NSPCs are present even after injury. Thus, to estimate the density of proliferating and differentiating NSPC in DG, we divided cell number to the DG volume including hilus and GCL but excluding ML.

The volume of the hilus is much bigger than the GCL volume in general. Therefore, the whole DG volume (GCL+hilus) is not significantly reduced after injury although the GCL volume seems to be reduced as shown in Supplementary Figure 2b, 2d and 2f. However, as the hilus volume remains unchanged the whole DG volume remains the same.
